# Supplementary material for: Copper–cobalt double metal cyanides as green catalysts for phosphoramidate synthesis
Source: Commun Chem. 2023 Jul 5;6:141. doi: 10.1038/s42004-023-00927-0 (PMC10322982; doi:10.1038/s42004-023-00927-0)
Supplement: Supplementary file 2 — Supplementary Information [file 42004_2023_927_MOESM2_ESM.pdf]

Supplementary Information

Copper-cobalt double metal cyanides as green catalysts for phosphoramidate synthesis

Alejandro Fonseca,[a,b] Aram L. Bugaev,[c,d] Anna Yu. Pnevskaya,[c] Kwinten Janssens,[a] Carlos Marquez,[a,*] Dirk De Vos.[a,*]

[a] Centre for Membrane Separations, Adsorption, Catalysis and Spectroscopy for Sustainable Solutions, KU Leuven, Celestijnenlaan 200F, 3001 Leuven, Belgium.

[b] Department of Polymer Engineering and Science, Polymer Processing, Montanuniversitaet Leoben, Otto Gloeckel-Strasse 2, 8700 Leoben, Austria.

[c] The Smart Materials Research Institute, Southern Federal University, Sladkova 178/24, Rostov-on-Don, 344090, Russia

[d] Paul Scherrer Institute, 5232 Villigen PSI, Switzerland

* Corresponding authors: carlos.marquez@kuleuven.be, dirk.devos@kuleuven.be

Supplementary Methods:

Synthesis of Cu based catalysts:

- Cu-BTC (MOF-199, copper(II)-benzene-1,3,5-tricarboxylate) was purchased from Sigma Aldrich.

- Cu supported on silica (Cu/SiO_2): 400 mg of CuI (2 mmol) were dissolved in 100 ml of ACN. To this solution, 1g of silica gel (pore size 60 Å, 230-400 mesh particle size, 40-63 µm particle size, Supelco) previously dried at 150°C was added, while magnetically stirring. This solution was left to evaporate at room temperature. When the solvent was completely evaporated, the catalyst was washed 3 times with ACN. The catalyst was then dried in the vacuum oven at 100°C.

- Cu-Mg-Al mixed oxide ($Cu_{0.33}Mg_{0.33}Al_{0.33}CO_3$): 4.8g of $Cu(NO_3)_2 \cdot 3\ H_2O$, 5 g of $Mg(NO_3)_2 \cdot 6\ H_2O$, and 7.42 g of $Al(NO_3)_3 \cdot 9\ H_2O$ were dissolved in 125 ml distilled water and transferred into a 250 ml five-necked flask equipped with magnetic agitation, reflux condenser and pH meter. This system was set at 60°C under vigorous stirring. Subsequently, 75 to 125 ml of a 0.75 M Na_2CO_3 aqueous solution was slowly added to the metal nitrate solution over 2 h until a pH of 8 was reached. The light blue suspension was stirred for 15h at room temperature and filtered. The recovered solid was intensively washed with distilled water and dried at 90°C *in-vacuo* for 24 h. The obtained samples were calcined for 4 h at 200°C.

Supplementary notes, figures and tables:

X-ray diffraction:

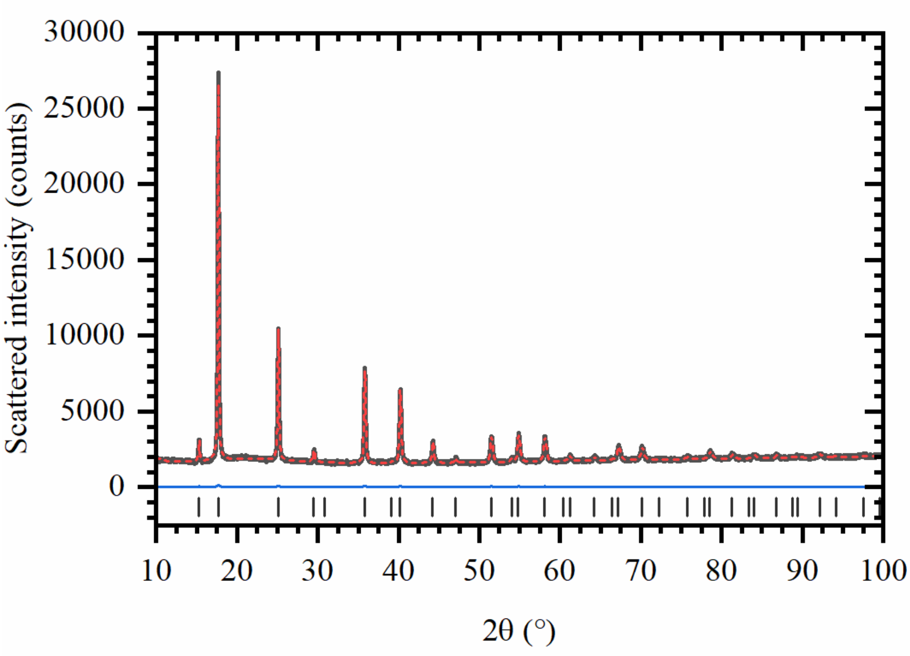

Fig. S1. Pawley refinement of Cu-Co DMC: observed pattern (+), calculated pattern (black line), difference (blue line) and expected peak positions (|).

Table S1. Crystallographic parameters obtained from the Pawley refinement of Cu-Co DMC.

	Cu-Co DMC
Space group	*Fm*-3*m*
a (Å)	10.0360(1)
R_{wp} (%)	2.69
GOF	1.20

N_2 physisorption:

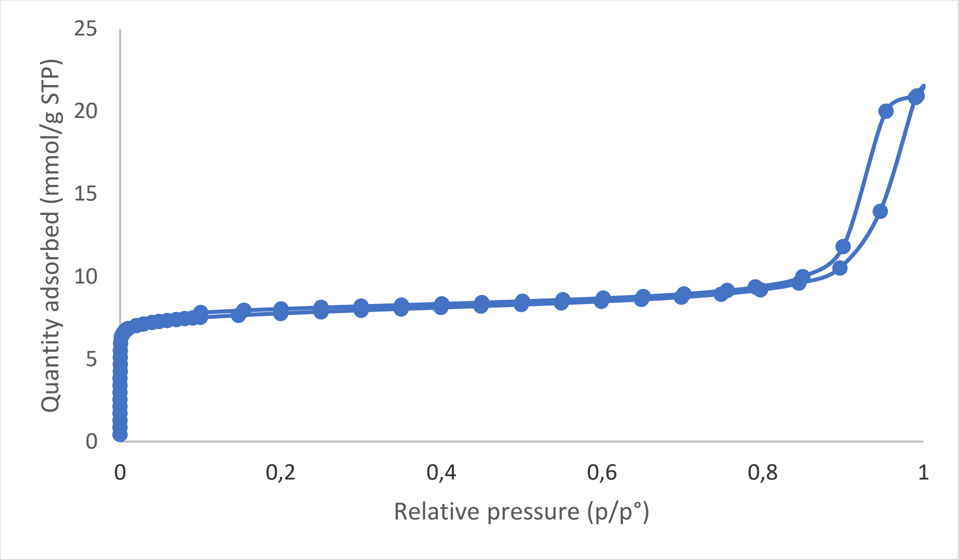

Fig. S2. Nitrogen physisorption isotherms of Cu-Co DMC.

Table S2. Textural properties of selected PBA samples determined from nitrogen physisorption.

Sample	S_{BET} (m^2/g)	S_{ext} (m^2/g)	V_{micro} (cm^3/g)	LAS (mmol/g)[a]
Cu-Co DMC	659	116	0.217	0.044

Inductively coupled plasma atomic emission spectroscopy:

Table S3. Elemental analysis of Cu and Co ions in the Cu-Co DMC catalyst.

Sample	Cu:Co ratio
Cu-Co DMC	1.7

Table S4. Elemental analysis of Cu ions remaining in solution after reaction.

Sample	Cu mol%[a]
Cu-BTC	> 99%
Cu-Co DMC	1.6%

[a] Total amount of Cu that leached from the solid catalyst.

Fourier-transform infrared spectroscopy:

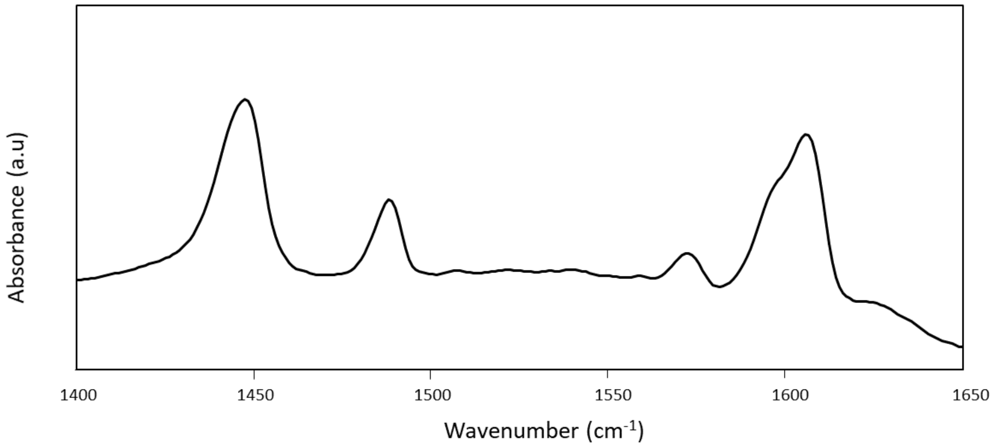

Fig. S3. IR spectra of adsorbed pyridine on Cu-Co DMC (amplified 1400-1650 cm^{-1} region).

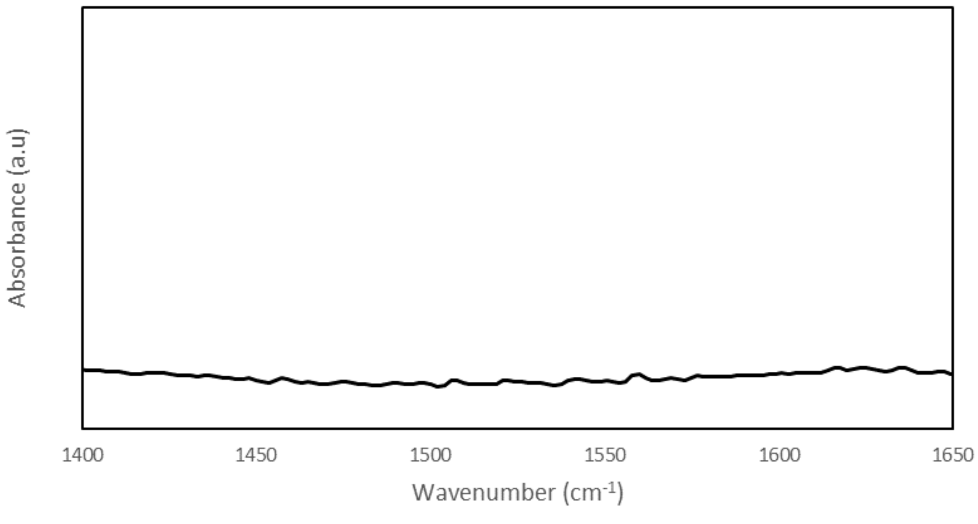

Fig. S4. IR spectra of Cu-Co DMC before adsorption of pyridine (amplified 1400-1650 cm^{-1} region).

Thermogravimetric analysis:

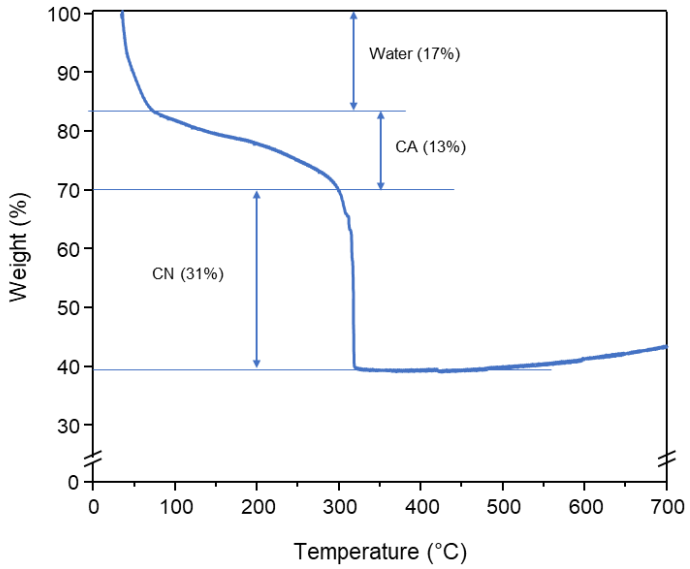

Fig. S5. Thermogravimetric analysis of Cu-Co DMC. CA = Complexing agent (*tert*-butanol). CN = Cyanide group.

Extended reaction optimization:

Table S5 Extended reaction optimization[a,b]

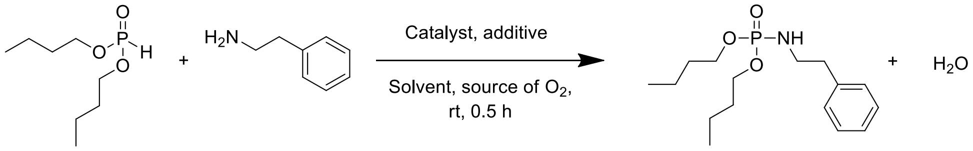

Entry	Catalyst (mol%)	Solvent	Phosphite/Amine/I_2 (equivalents)	Source of O_2	Yield
1	CuI (20%)	ACN	1/2/0	Air	73% [c]
2	$Cu(OAc)_2$ (20%)	ACN	1/2/0	Air	4% [c]
3	CuO (20%)	ACN	1/2/0	Air	>1%[c]
4	Cu BTC (3%)	ACN	1/2/0	Air	2% [c]
5	Cu/SiO_2 (3%)	ACN	1/2/0	Air	>1% [c]
6	$Cu_{0.33}Mg_{0.33}Al_{0.33}CO_3$ (3%)	ACN	1/2/0	Air	11% [c]
7	$Cu(OAc)_2$ (20%)	ACN	1/2/0.20	Air	86% [c]
8	$Cu(OAc)_2$ (20%)	ACN	1/2/0.20	O_2 balloon	90%
9	Cu BTC (3%)	DCM	1/2/0.15	O_2 balloon	98%
10	CuO	DCM	1/2/0.15	O_2 balloon	20%
11	Cu/SiO_2 (3%)	DCM	1/2/0.15	O_2 balloon	12%
12	$Cu_{0.33}Mg_{0.33}Al_{0.33}CO_3$	DCM	1/2/0.15	O_2 balloon	35%

[a] All reactions were performed using dibutyl phosphite as limiting reagent at a scale of 2 mmol, amine, catalyst, solvent (4 ml), iodine and a source of oxygen at room temperature for half an hour. [b] Yields of dibutyl phenylethyl phosphoramidates were determined by ^{1}H NMR spectroscopy using 1,3,5-trimethoxybenzene as internal standard. [c] Reaction time 3 h. Cu-BTC: MOF-199, copper(II)-benzene-1,3,5-tricarboxylate), Cu supported on silica: Cu/SiO_2 and Cu-Mg-Al mixed oxide: $Cu_{0.33}Mg_{0.33}Al_{0.33}CO_3$.

Hot Filtration Test :

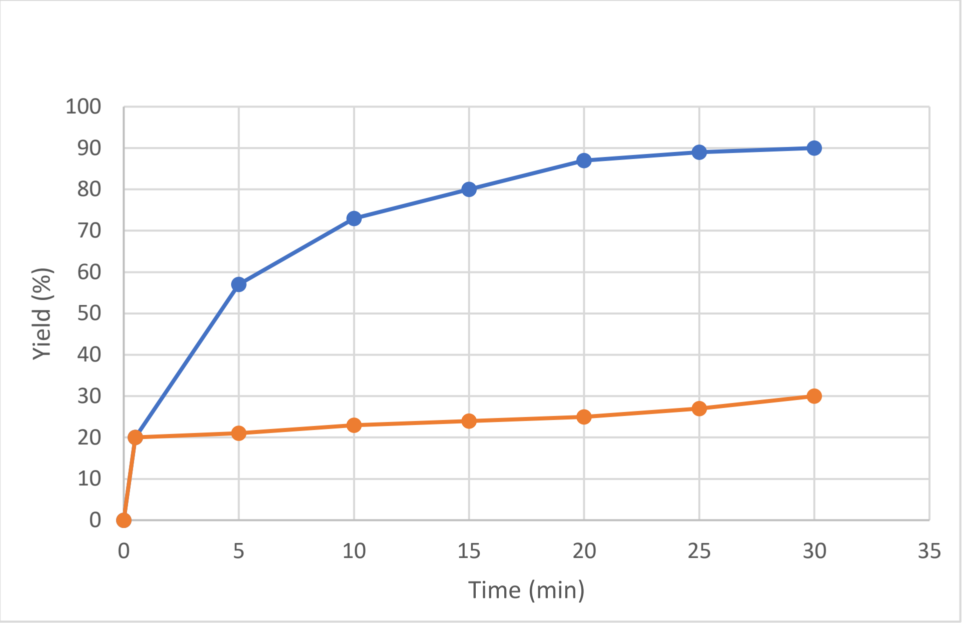

Fig. S6. Hot filtration test of optimized model reaction: dibutyl phosphite (2 mmol) and phenethylamine (4 mmol) at room temperature in DCM, 40 mg of Cu-Co DMC, 75 mg of I_2 and O_2 balloon.

Recycling tests:

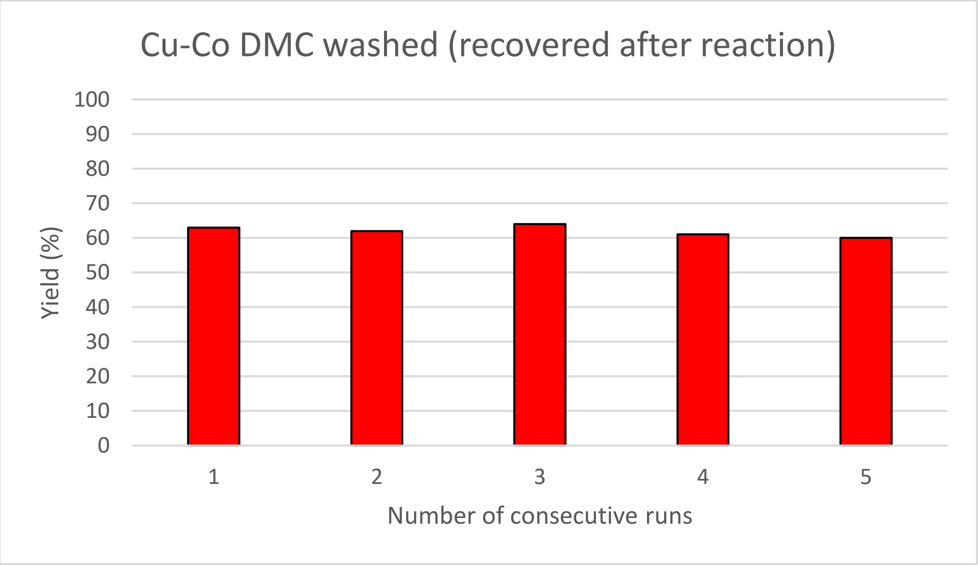

Fig. S7. Recycling tests for the coupling of dibutyl phosphite (2 mmol) and phenethylamine (4 mmol) at room temperature in DCM, 40 mg of Cu-Co DMC, 75 mg of I_2 and O_2 balloon. Catalyst was washed after each run using excess methanol and excess water:*tert*-butanol mixture. Yield of phosphoramidate after 15 min of reaction time.

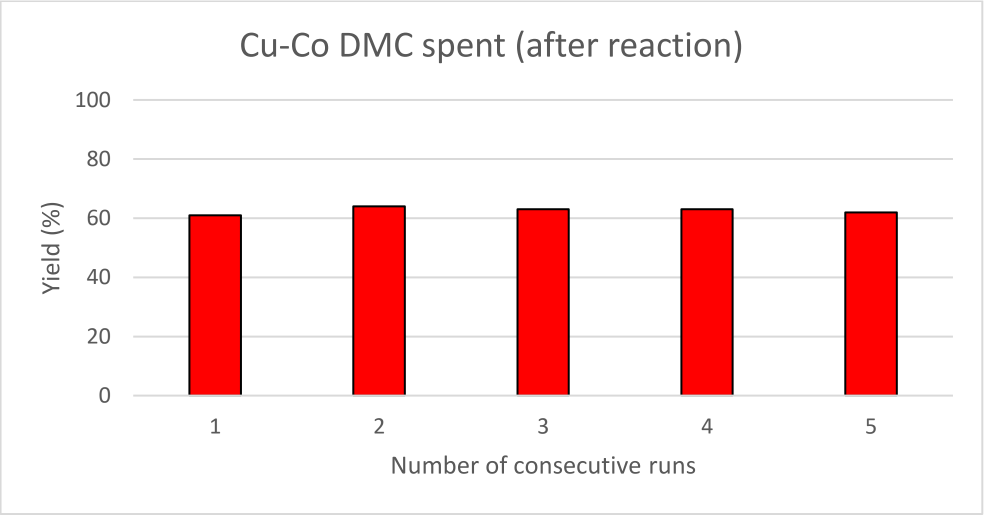

Fig. S8. Recycling tests for the coupling of dibutyl phosphite (2 mmol) and phenethylamine (4 mmol) at room temperature in DCM, 40mg of Cu-Co DMC (spent), 75 mg of I_2 and O_2 balloon. Catalyst was centrifugated and used as such after each consecutive run. Yield of phosphoramidate after 15 min of reaction time.

UV-Vis measurements:

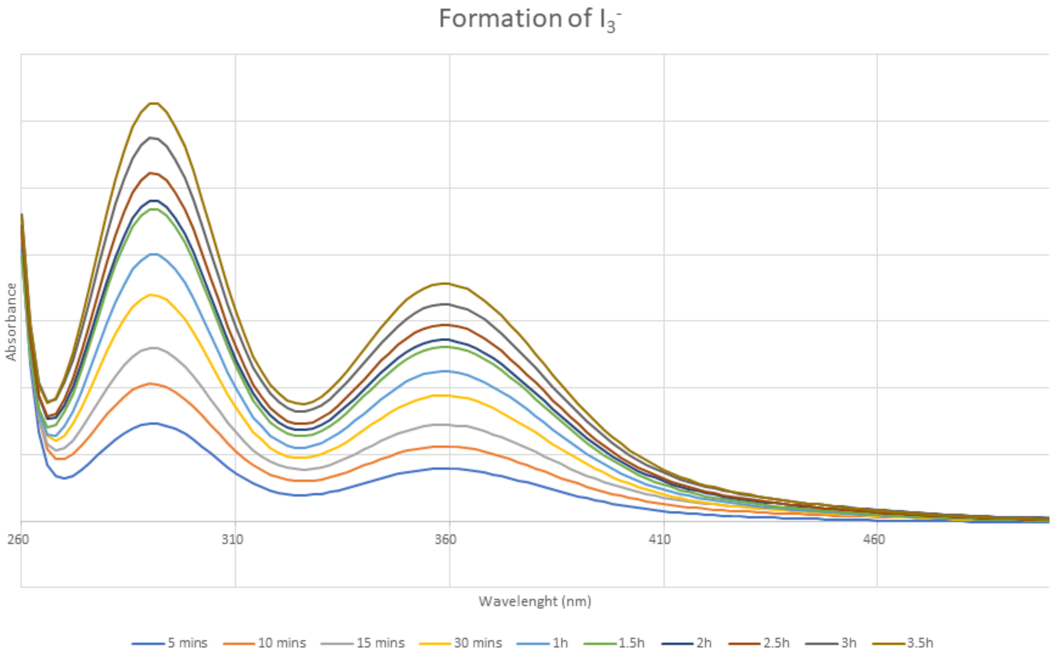

Fig. S9. UV/Vis spectra of I_3^- at different reaction times. Reaction condition: NaI (0.6 mmol), HCl (75 mM), Cu-Co DMC (40 mg), in methanol, opened to air.

Supplementary Note 1: Detection of I_2 in solution was achieved by measurement of the UV-Vis spectral signal of I_3^- at 360 nm. This was done by diluting the desired I_2 containing sample (to $5x10^{-3}$ mM - $1x10^{-2}$ mM) in a 100mM NaI solution, transforming all I_2 into I_3^-. Extrapolation of the concentration of I_2 was done using a previously constructed calibration curve.

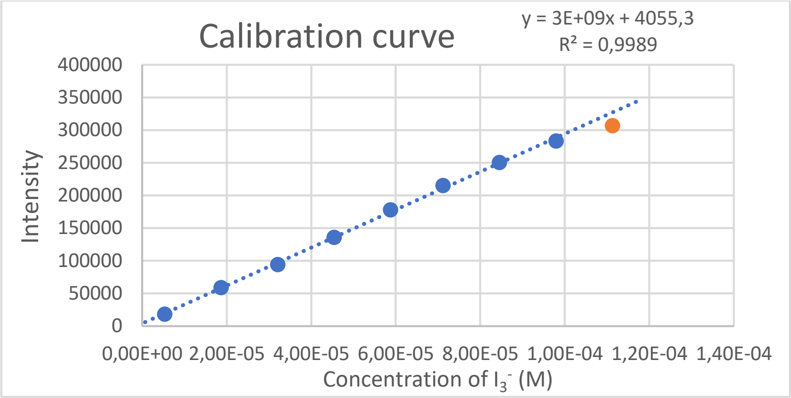

Fig. S10. Calibration curve for the quantification of I_3^- in methanolic solution.

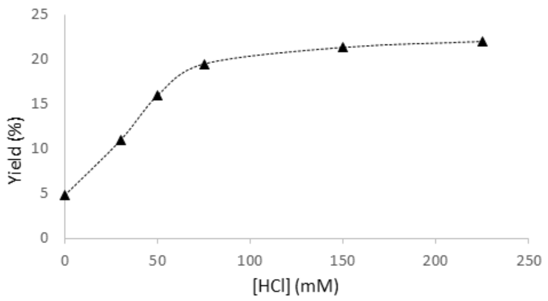

Fig. S11 I_2 yield in the iodide oxidation by Cu-Co DMC in methanolic solution vs HCl concentration after 2.5 h reaction time. 2 mmol of NaI, room temperature and exposed to air. Yields were determined by measuring I_3^- via UV-Vis measurements (λ = 360 nm)

Supplementary Discussion 1: The overall low rate of oxidation of I^- to I_2, compared to the rate observed in the model reaction for the coupling of amines and phosphites could be explained by the use of the more polar solvent MeOH, instead of DCM. As in the case of ACN, a polar solvent such as methanol is presumably able to interact with the Lewis acid catalytic sites of the Cu-Co DMC, hindering the reaction. It is also worth noting that while the changes in oxidation-reduction potential due to the addition of HCl to the reaction are not the same when using different solvents, it helps us discern any underlying trend in the transformation.

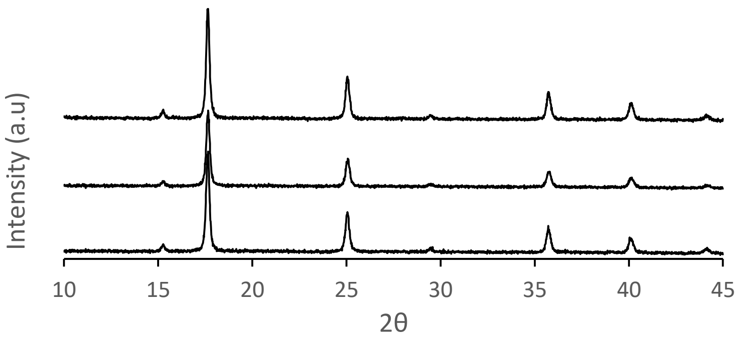

Fig. S12. X-ray diffractograms of the Cu-Co DMC catalyst after 24h in methanolic solutions of different concentration of HCl: **top** 75 mM, **middle** 150 mM, **bottom** 225 mM.

XANES and EXAFS analyses:

Supplementary Note 2: In the following, theoretical XANES spectra were calculated in FDMNES code within the finite difference method. The convolution of theoretical spectra was performed using PyFitIt code. Optimization of structural parameters (interatomic distances) was also performed in PyFitIt code using machine learning approach.

Supplementary Note 3: EXAFS fitting was performed in the Artemis program of Demeter package. For the data presented in Figure 2c,d of the main text, the crystallographic model of Co-Cu cyanide was taken and the fitting was performed for the first three coordination shells (up to metal-metal contribution) including also the multiple scattering contributions. Due to limited N_{ipd} given by the limited *k*-range, the following assumptions were made for the 3-shell fitting: all coordination numbers were fixed to 6, zero energy shift parameter was common for all contributions, and Debye-Waller parameter of multiple scattering paths were equal to that of the corresponding single scattering path. For accurate analysis of the first-shell coordination numbers, the fitting range was limited to 1..2 Å, and the fitting was performed varying 4 parameters for the first shell contribution: coordination number (*N*), interatomic distance (*R*), Debye-Waller parameter (σ^2), and zero energy shift (ΔE_0), and then fixing ΔE_0.

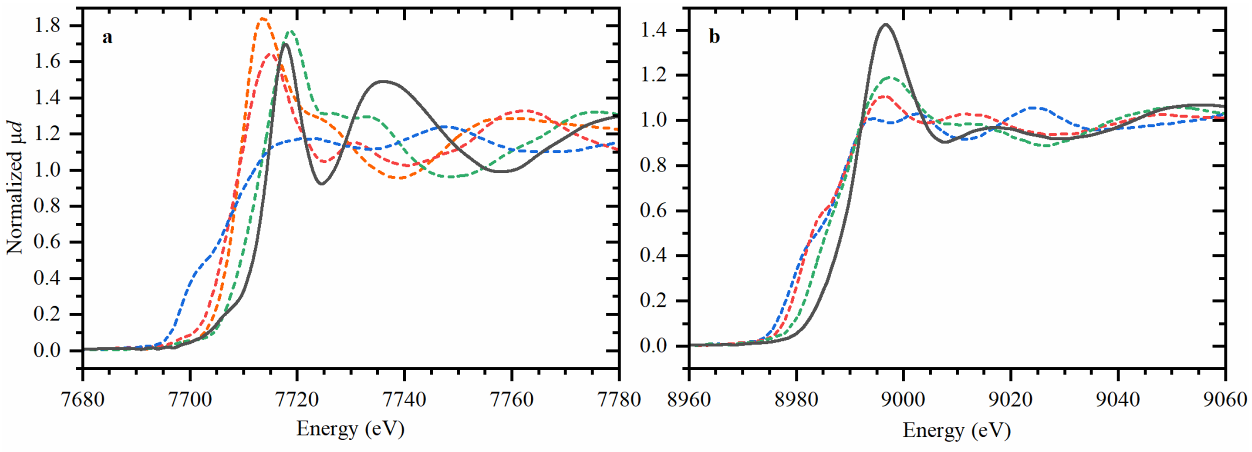

Fig. S13. Experimental XANES spectra for the fresh catalyst (solid black lines) in comparison with reference samples (dashed colored lines). (a) Co foil (blue), $Co(acac)_2$ (orange), CoO (red), and $Co(OH)_3$ (green). (b) Cu foil (blue), Cu_2O (red), and CuO (green).

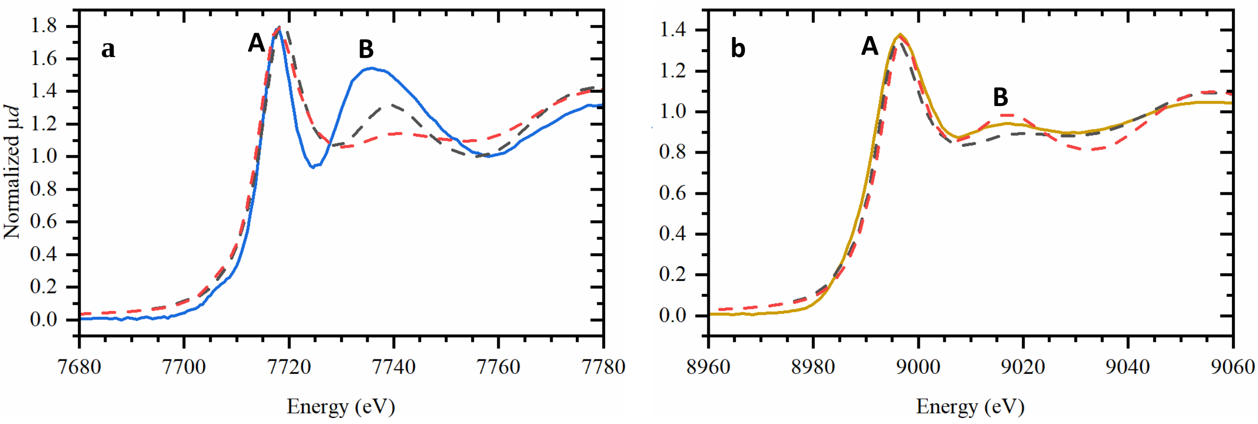

Fig. S14. Experimental (solid lines) and theoretical (dashed lines) XANES spectra for Cu-C-N-Co alignment (dashed red) and Cu-N-C-Co alignment (dashed black). In particular, reverse alignment (Cu-C-N-Co) of the CN-ligand results in the underestimated curvature of the maximum B and adjacent minima at Co *K*-edge (a), and overestimated curvature of the corresponding features at Cu *K*-edge (b). Interatomic distances were kept fixed.

Table S6. The results of the first shell fitting of the initial state of the catalyst.

Scattering path	*N*	*R* (Å)	σ^2 (Å^2)	ΔE_0 (eV)
Co-C	6.2 ± 2.8	1.92 ± 0.01	0.004 ± 0.011	1.4
Cu-N	4.0 ± 1.1	2.00 ± 0.01	0.007 ± 0.004	0

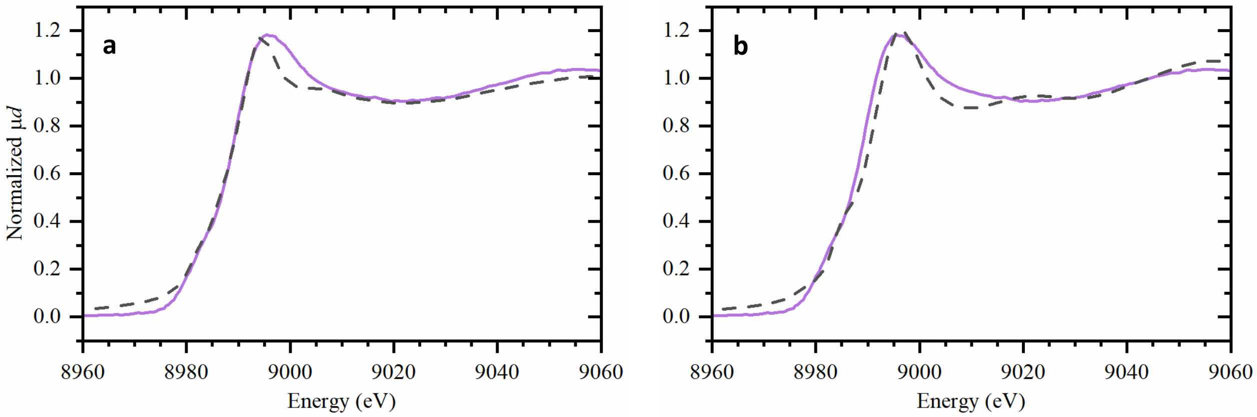

Figure S15. Experimental Cu *K*-edge XANES spectra of the spent catalyst after reaction (solid purple lines), and theoretical spectra (dashed black) for square planar Cu sites coordinated by -OH (a) and -NC (b) ligands. The interatomic distances were optimized to obtain the best agreement with the experimental data. The first model gives slightly better agreement with the experimental spectrum. However, due to a big variety of hypothetical structures and possibility for their mixtures, Cu local structure in the spent catalyst was characterized as square planar Cu–X (X = N, O).

Product identification:

Dibutyl phenylethylphosphoramidate:

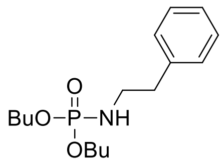

^{1}H NMR (400 MHz, $CDCl_3$): δ = 7.35-7.15 (m, 5 H), 4.00-3.86 (m, 4 H), 3.43 (s, 1H), 3.22-3.10 (m, 2 H), 2.80 (t, J = 8.00 Hz, 2 H), 1.69-1.57 (m, 4 H), 1.46-1.32 (m, 4 H), 0.93 (t, J = 7.34 Hz, 6 H)

^{13}C NMR (100 MHz): δ = 133.7, 126.8, 126.2, 125.8, 65.3, 39.6, 33.2, 27.5, 17.2, 14.3

^{31}P NMR: (161 MHz, $CDCl_3$): δ = 8.97

GC/MS (EI, 70 eV): Calcd for $C_{16}H_{28}NO_3P^+$ $[M]^+$ requires 313.37; found 313.10

Dibutyl propylphosphoramidate:

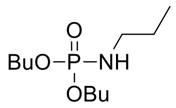

^{1}H NMR (400 MHz, $CDCl_3$): δ = 5.56 (s, 1H), 4.03-3.94 (m, 4 H), 2.90 (t, J = 7.44, 2 H), 1.66 (m, 4 H), 1.52 (m, 2 H), 1.41 (m, 4 H), 1.00 (t, J = 7.44, 3 H), 0.95 (t, J = 7.32, 6 H)

^{13}C NMR (100 MHz): δ = 65.2, 38.3, 25.3, 21.1, 19.5, 13.2, 11.4

^{31}P NMR: (161 MHz, $CDCl_3$): δ = 9.20

GC/MS (EI, 70 eV): Calcd for $C_{11}H_{26}NO_3P^+$ $[M]^+$ requires 251,30; found 251.20.

Dibutyl allylphosphoramidate:

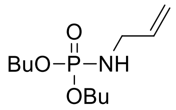

^{1}H NMR (400 MHz, $CDCl_3$): δ = 5.90-5.88 (m, 1H), 5.20 (dd, J = 11.16, 1.21, 1H), 5.11 (dd, J = 10.4, 1.30, 1 H), 4.02-3.99 (m, 4 H), 3.85-3.82 (m, 2 H), 3.68 (s, 1 H), 1.69-1.67 (m, 4 H), 1.47-1.43 (m, 4 H), 0.95 (t, J = 7.40, 6 H)

^{13}C NMR (100 MHz): δ = 130.6, 120.2, 63.4, 48.1, 25.2, 17.1, 14.1

^{31}P NMR: (161 MHz, $CDCl_3$): δ = 8.68

GC/MS (EI, 70 eV): Calcd for $C_{11}H_{24}NO_3P^+$ $[M]^+$ requires 249.29; found 249.20.

Dibutyl piperidinphosphoramidate:

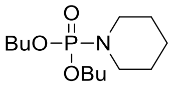

^{1}H NMR (400 MHz, $CDCl_3$): δ = 3.94-3.91 (m, 4 H), 3.10-3.07 (m, 2 H), 2.78 (t, J = 7.26, 2H), 1.66-1.62 (m, 4 H), 1.54-1.51 (m, 6 H), 1.40-1.37 (m, 4 H), 0.90 (t, J = 7.40, 6 H)

^{13}C NMR (100 MHz): δ = 63.2, 40.4, 32.3, 26.5, 22.1, 18.4, 14.2

^{31}P NMR: (161 MHz, $CDCl_3$): δ = 8.26

GC/MS (EI, 70 eV): Calcd for $C_{13}H_{28}NO_3P^+$ $[M]^+$ requires 277.34; found 277.10

Dibutyl benzylphosphoramidate:

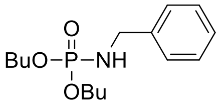

^{1}H NMR (400 MHz, $CDCl_3$): δ = 7.39-7.35 (m, 5H), 4.12-4.09 (m, 6 H), 3.67 (s, 1H) 1.63 -1.59 (m, 4 H), 1.38-1.35 (m, 4 H), 0.93 (t, J = 7.37, 6 H)

^{13}C NMR (100 MHz): δ = 140.1, 139.8, 139.6, 139.4, 63.1, 48.7, 32.4, 22.2, 14.3

^{31}P NMR: (161 MHz, $CDCl_3$): δ = 7.77

GC/MS (EI, 70 eV): Calcd for $C_{15}H_{26}NO_3P^+$ $[M]^+$ requires 299.35; found 299.10

Dibutyl isopropilphosphoramidate:

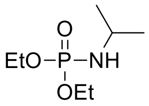

^{1}H NMR (400 MHz, $CDCl_3$): δ = 3.89-3.72 (m, 4 H), 3.30-3.26 (m, 1H), 3.10 (s, 1 H), 1.22 (t, J = 7.28, 6 H), 1.07 (d, J = 6.32, 6H)

^{13}C NMR (100 MHz): δ = 63.1, 42.8, 30.7, 21.2, 18.7

^{31}P NMR: (161 MHz, $CDCl_3$): δ = 8.72

GC/MS (EI, 70 eV): Calcd for $C_7H_{18}NO_3P^+$ $[M]^+$ requires 195,20; found 195.30

Diethyl phenylethylphosphoramidate:

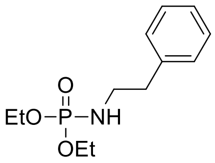

^{1}H NMR (400 MHz, $CDCl_3$): δ = 7.35-7.19 (m, 5 H), 4.08-3.93 (m, 4 H), 3.23-3.13 (m, 2 H), 3.02 (s, 1H), 2.81 (t, J = 6.88, 2 H), 1.30 (t, J = 7.08, 6 H)

^{13}C NMR (100 MHz): δ = 130.8, 129.1, 128.7, 127.5, 63.4, 42.1, 28.9, 15.2

^{31}P NMR: (161 MHz, $CDCl_3$): δ = 9.54

GC/MS (EI, 70 eV): Calcd for $C_{12}H_{20}NO_3P^+$ $[M]^+$ requires 257.27; found 257.30

Dimethyl phenylethylphosphoramidate:

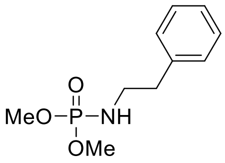

^{1}H NMR (400 MHz, $CDCl_3$): δ = 7.34-7.18 (m, 5 H), 3.63-3.54 (m, 6 H), 3.19-3.10 (m, 2 H), 2.93 (s, 1 H) 2.83, (t, J = 7.08, 2 H)

^{13}C NMR (100 MHz): δ = 131.2, 128.7, 127.9, 127.6, 52.1, 38.4, 25.4

^{31}P NMR: (161 MHz, $CDCl_3$): δ = 11.43

GC/MS (EI, 70 eV): Calcd for $C_{10}H_{16}NO_3P^+$ $[M]^+$ requires 229.21; found 229.40

Dimethyl benzoylphosphoramidate:

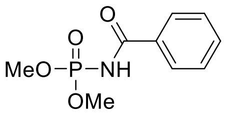

^{1}H NMR (400 MHz, $CDCl_3$): δ = 7.90-7.85 (m, 2 H), 7.54-7.40 (m, 3 H), 5.10 (s, 1 H), 3.70-3.58 (m, 6 H)

^{13}C NMR (100 MHz): δ = 180.5, 132.1, 129.1, 128.4, 127.9, 60.8

^{31}P NMR: (161 MHz, $CDCl_3$): δ = 12.20

GC/MS (EI, 70 eV): Calcd for $C_9H_{12}NO_4P^+$ $[M]^+$ requires 229,17; found 229.00

Dimethyl phenylphosphoramidate:

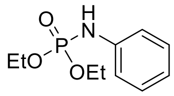

^{1}H NMR (400 MHz, $CDCl_3$): δ = 7.36 (t, J = 8.3, 2 H), 7.25 – 7.22 (m, 2 H), 6.83 (tt, J = 7.1, 1.2, 1 H), 6.71 (d, J = 8.7, 1 H), 3.89 – 3.83 (m, 4 H), 1.30 (td, J = 7.1, 0.6, 6 H)

^{13}C NMR (100 MHz): δ = 139.2, 128.3, 122.1, 116.7, 62.4, 16.2

^{31}P NMR: (161 MHz, $CDCl_3$): δ = 8.52

GC/MS (EI, 70 eV): Calcd for $C_{10}H_{16}NO_3P^+$ $[M]^+$ requires 229.22; found: 229.30

Diethyl (4-ethoxyphenyl)phosphoramidate:

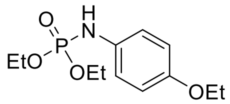

^{1}H NMR (400 MHz, $CDCl_3$): δ = 7.54 (d, J = 8.8, 1 H), 7.20 (d, J = 8.7, 2 H), 6.98 (d, J = 8.6, 2H), 4.09-4.05 (m, 6 H), 1.34 (t, J = 8.4, 3 H), 1.20 (t, J = 8.1)

^{13}C NMR (100 MHz): δ = 152.0, 134.1, 116.9, 115.2, 64.6, 62.1, 16.0, 14.8

^{1}P NMR: (161 MHz, $CDCl_3$): δ = 7.43

GC/MS (EI, 70 eV): Calcd for $C_{12}H_{20}NO_4P^+$ $[M]^+$ requires 273.27; found: 273.20

Diethyl 4(*tert*-butyl)phosphoramidate:

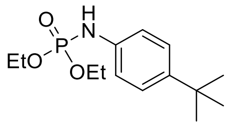

^{1}H NMR (400 MHz, $CDCl_3$): δ = 7.19 (d, J = 8.6, 2 H), 6.98 (d, J = 8.7, 2H), 4.08-4.04 (m, 4 H), 1.27 (s, 9 H), 1.24-1.20 (m, 6 H)

^{13}C NMR (100 MHz): δ = 141.3, 139.4, 128.5, 115.9, 62.1, 34.2, 31.3, 16.3

^{1}P NMR: (161 MHz, $CDCl_3$): δ = 7.48

GC/MS (EI, 70 eV): Calcd for $C_{14}H_{24}NO_3P^+$ $[M]^+$ requires 285,32; found: 285,40

Ethyl (diethoxyphosphoryl)-L-alaninate:

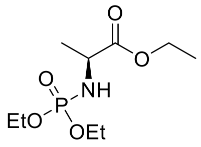

^{1}H NMR (400 MHz, $CDCl_3$): δ = 4.17-4.12 (m, 2H), 4.08-4.02 (m, 4H), 3.92-3.88 (m, 1H), 1.49 (d, J = 7.23, 3H), 1.30-1.22 (m, 9H)

^{13}C NMR (100 MHz): δ = 171.5, 62.1, 61.3, 46.6, 19.1, 16.0, 14.1

^{1}P NMR: (161 MHz, $CDCl_3$): δ = 7.40

GC/MS (EI, 70 eV): Calcd for $C_9H_{20}NO_5P^+$ $[M]^+$ requires 253,23; found: 253,50

Dimethyl (diethoxyphosphoryl)-L-aspartate:

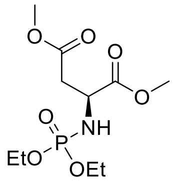

^{1}H NMR (400 MHz, $CDCl_3$): δ = 4.02-3.97 (m, 4 H), 3.92-3.89 (m, 1H), 3.61 (s, 3H), 3.56 (s, 3H), 3.09-3.04 (m, 2H), 1.23 (t, J = 7.33, 6H)

^{13}C NMR (100 MHz): δ = 171.6, 169.3, 62.3, 51.9, 44.9, 38.8, 15.8

^{1}P NMR: (161 MHz, $CDCl_3$): δ = 7.62

GC/MS (EI, 70 eV): Calcd for $C_{10}H_{20}NO_7P^+$ $[M]^+$ requires 297,24; found: 297,10

6-(benzylamino)dibenzo[c,e][1,2]oxaphosphinine 6-oxide:

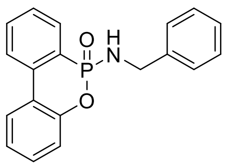

^{1}H NMR (400 MHz, $CDCl_3$): δ = 8.01 (d, J = 7.47, 1H), 7.75 (d, J = 7.51, 1H), 7.51-7.18 (m, 11H), 4.14 (d, J = 4.68, 1H), 4.06 (s, 2H)

^{13}C NMR (100 MHz): δ = 150.2, 141.6, 136.6, 136.4, 133.9, 132.8, 129.0, 128.5, 127.7, 126.8, 125.0, 121.9, 121.2, 119.9, 43.2.

^{1}P NMR: (161 MHz, $CDCl_3$): δ = 16.24

GC/MS (EI, 70 eV): Calcd for $C_{19}H_{16}NO_2P^+$ $[M]^+$ requires 321,32; found: 321,40

Supplementary Note 4

NMR spectra of final crude reaction mixture:

A typical ^{1}H-NMR spectrum of product mixture (i.e. oxidative coupling of dibutyl phosphite and propylamine) is shown in figure S19. To determine the concentration of the desired product (i.e. dibutyl propylphosphoramidate) a characteristic peaks of the compound was integrated using the Bruker TopSpin 4.1.3 software. With the use of an external standard (i.e. trimethoxybenzene) the final yield could be determined as follow:

$$Y(\%) = \frac{(\frac{P_{std.}}{P_{SM}} \times \frac{nH_{std.}}{nH_{Product}} \times \frac{A_{Product}}{A_{std.}} \times M_{std.})}{M_{SM}} \text{ x } 100$$

Where $P_{std.}$ = purity of the internal standard, P_{SM} = purity of the limiting reagent, $nH_{std.}$= number of protons corresponding to the selected standard peak, $nH_{Product}$ = number of protons corresponding to the selected product peak, $A_{Product}$ = area of the selected product peak, $A_{std.}$ = area of the selected standard peak, $M_{std.}$ = mols of standard and M_{SM} = mols of limiting reagent.

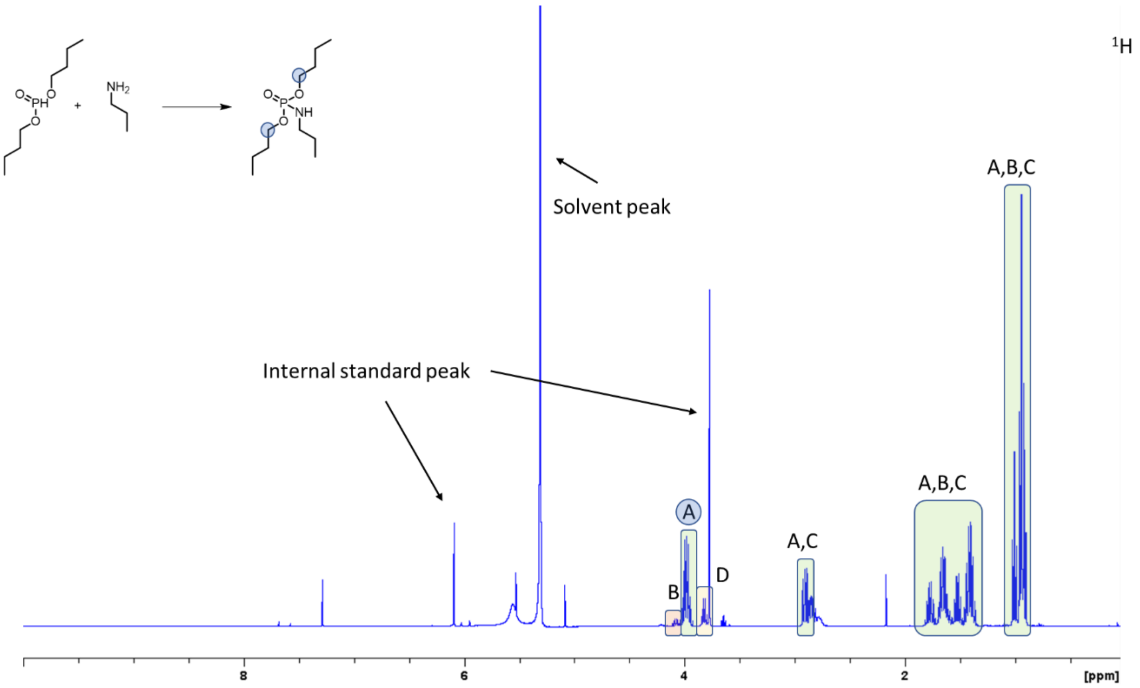

Figure S16: Typical ^{1}H-NMR spectrum of a product mixture. Signals corresponding to product and reagents are highlighted in a colored box. Selected signal for quantification is highlighted on a colored (blue) circle. Corresponding protons are also highlighted.

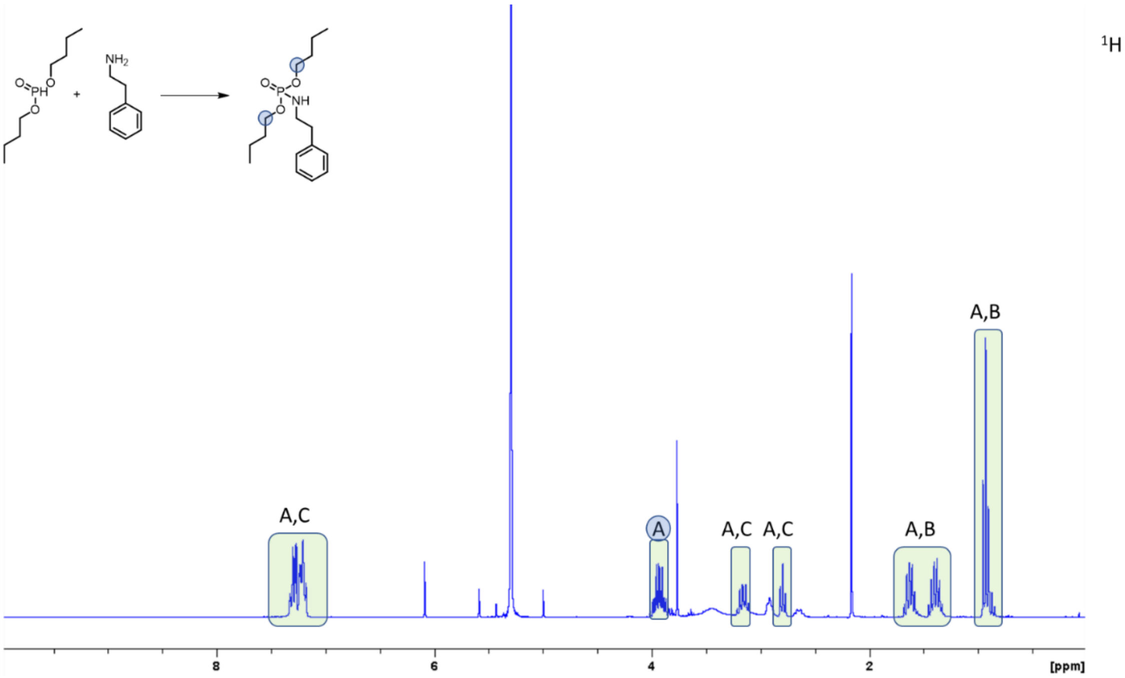

A: Dibutyl phenylethylphosphoramidate, B: Dibutyl phosphite, C: Phenylethylamine

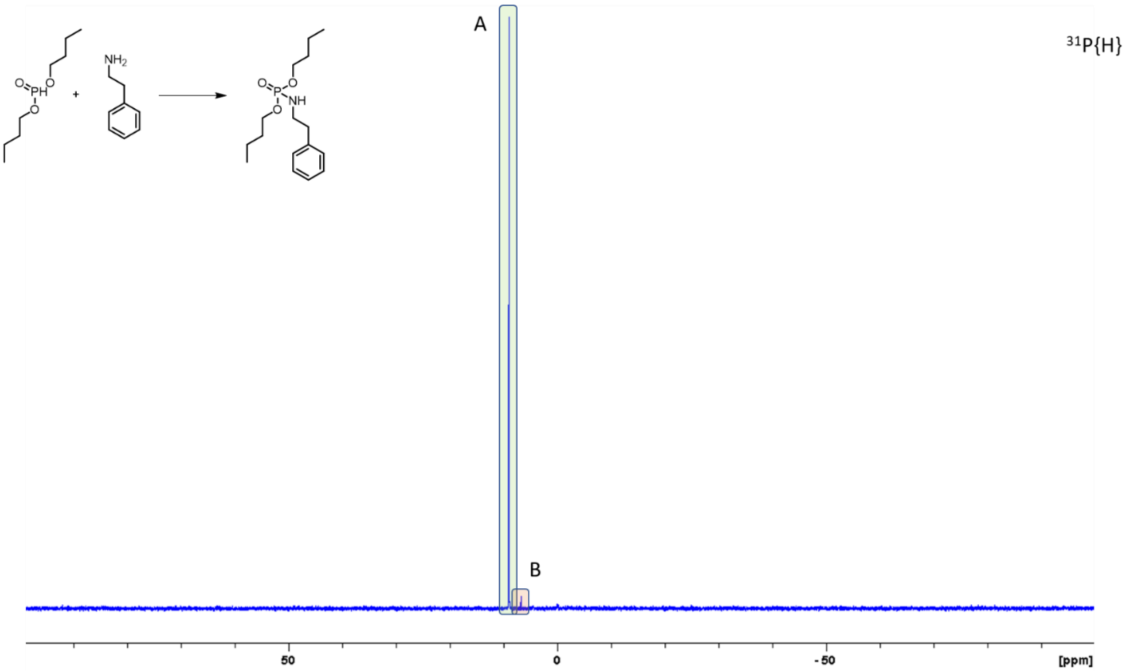

A: Dibutyl phenylethylphosphoramidate, B: Dibutyl phosphite

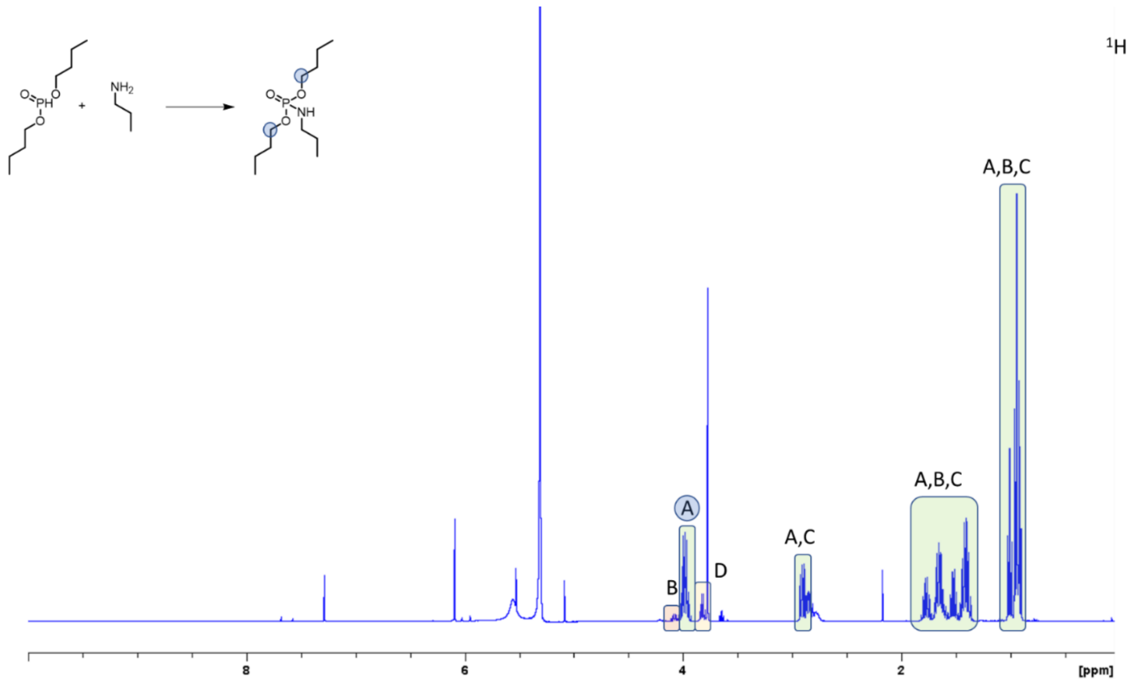

A: Dibutyl propylphosphoramidate, B: Dibutyl phosphite, C: Propilamine, D: Dibutyl phosphate

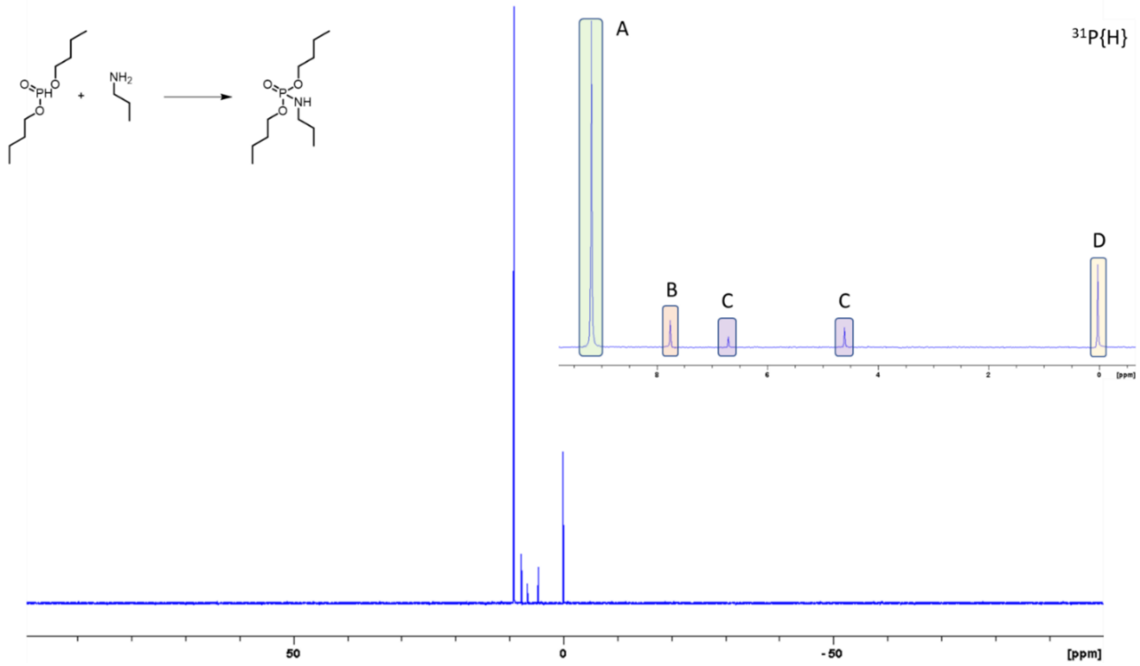

A: Dibutyl propylphosphoramidate, B: Dibutyl phosphite, C: Unknown, D: Dibutyl phosphate

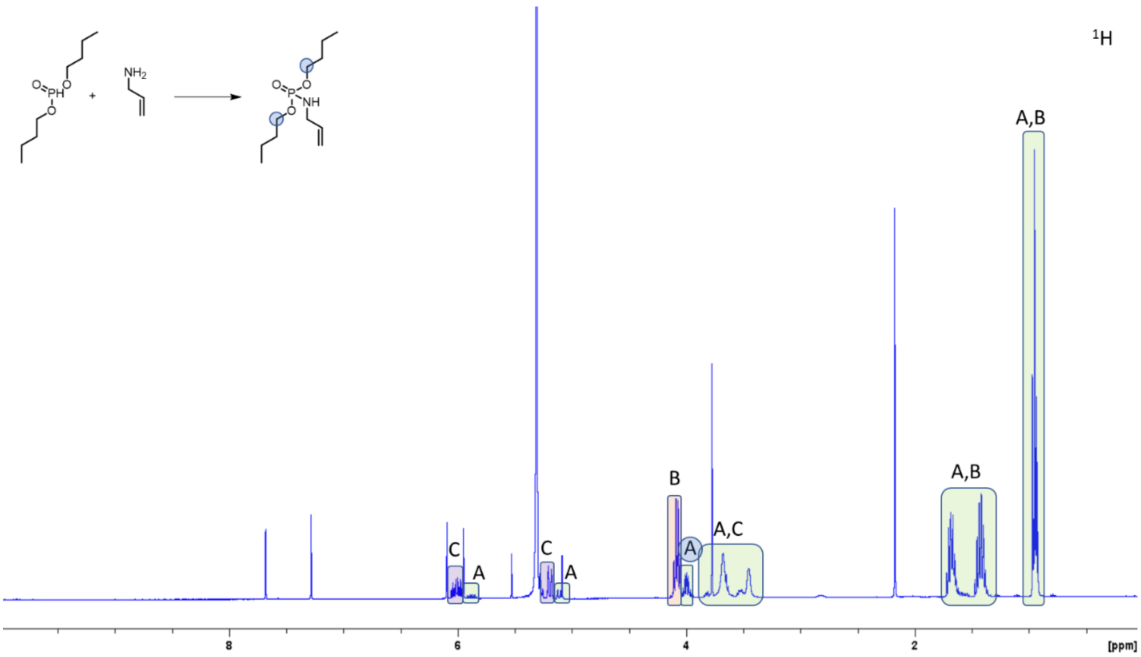

A: Dibutyl allylphosphoramidate, B: Dibutyl phosphite, C: Allylamine

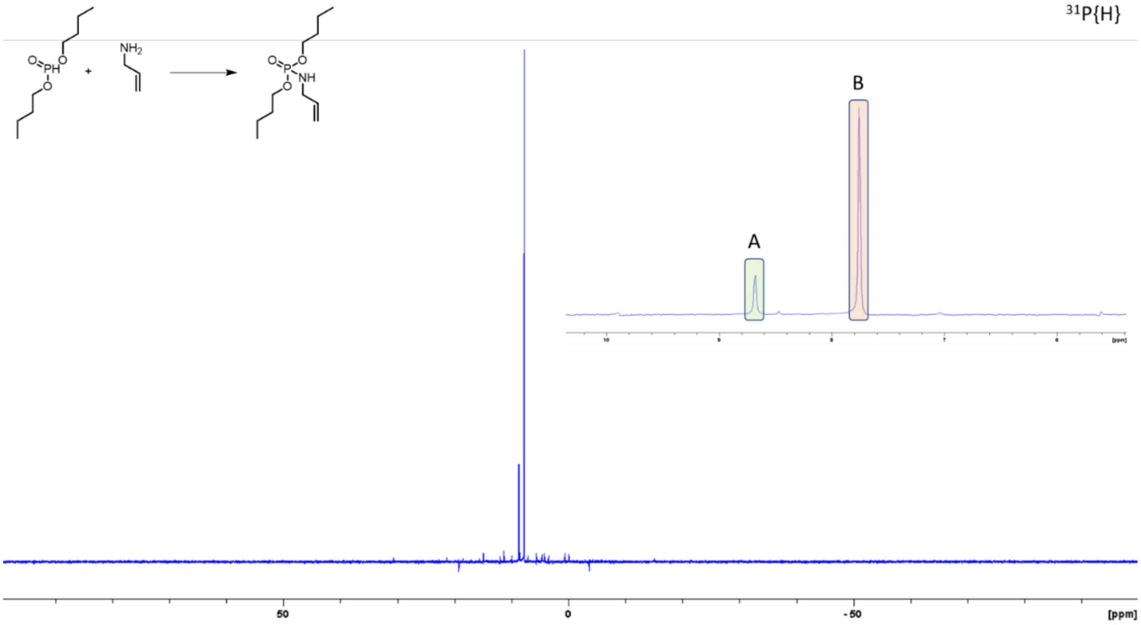

A: Dibutyl allylphosphoramidate, B: Dibutyl phosphite

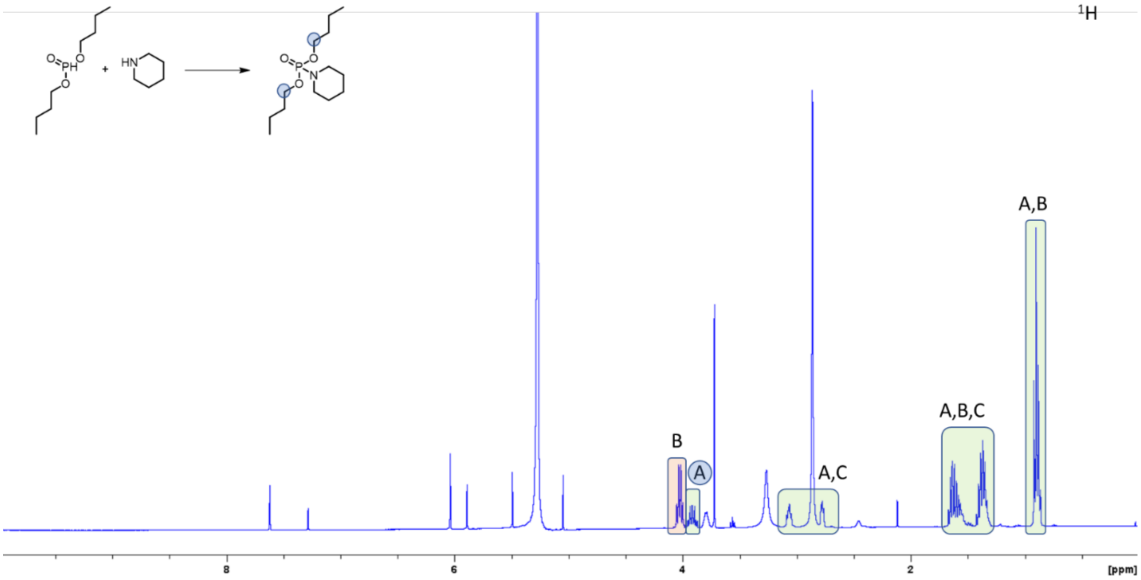

A: Dibutyl piperidinphosphoramidate, B: Dibutyl phosphite, C: Piperidine

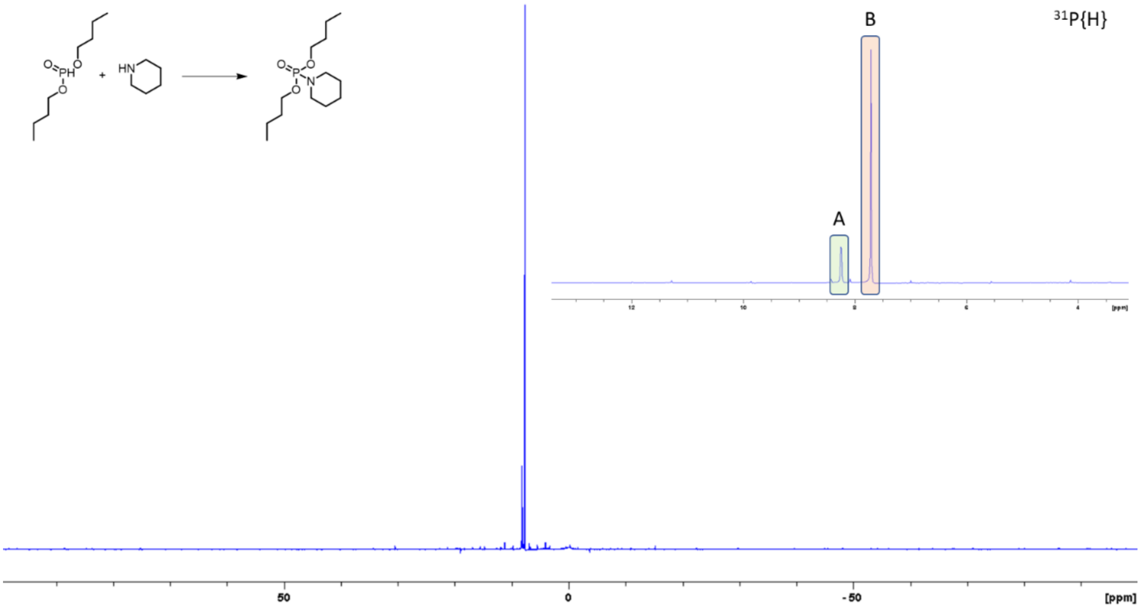

A: Dibutyl piperidinphosphoramidate, B: Dibutyl phosphite

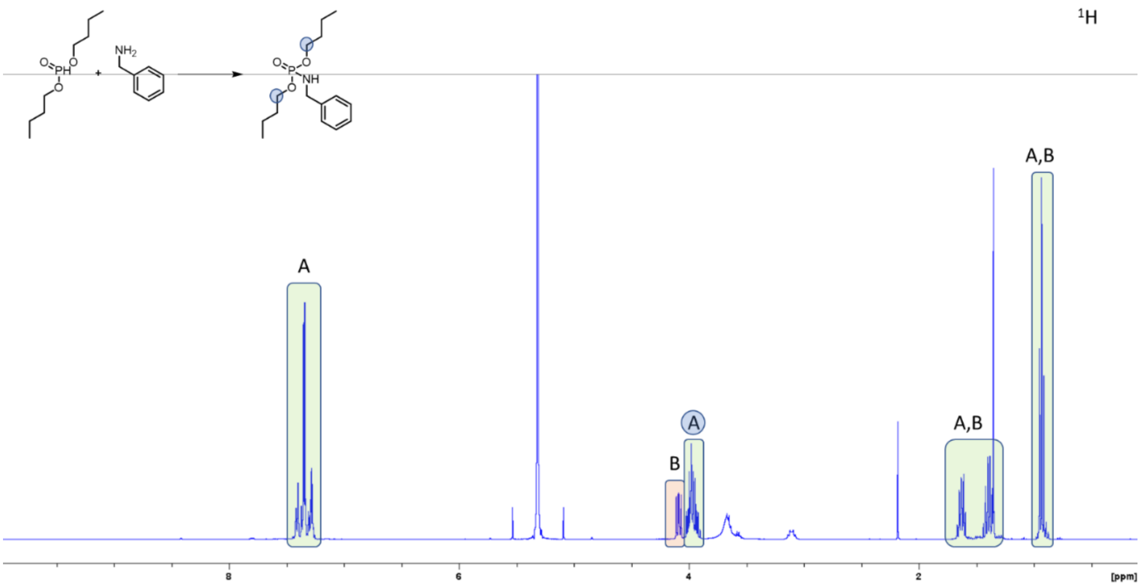

A: Dibutyl benzylphosphoramidate, B: Dibutyl phosphite

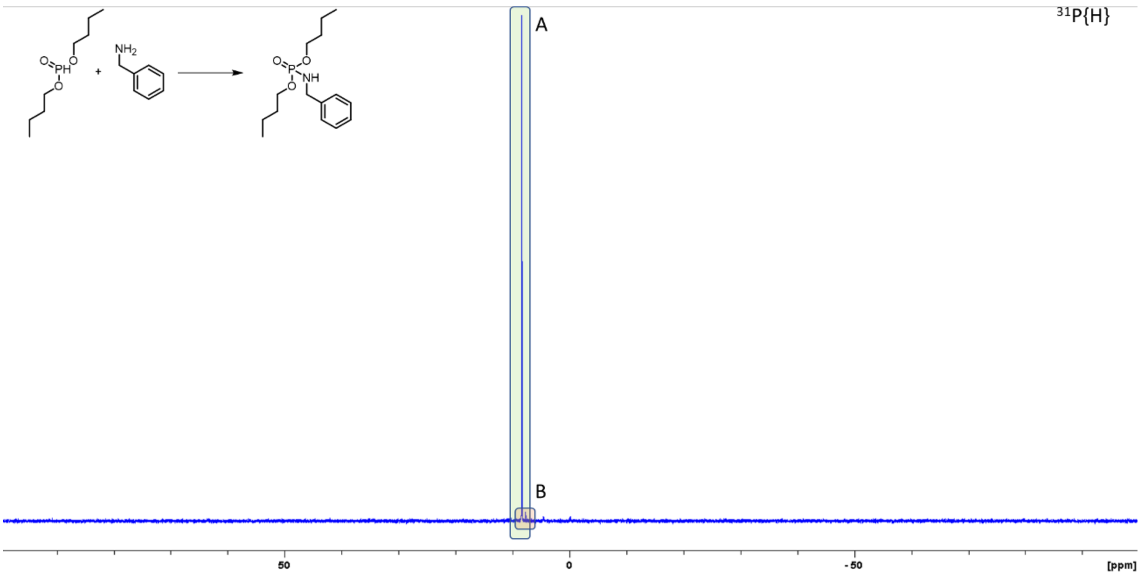

A: Dibutyl benzylphosphoramidate, B: Dibutyl phosphite

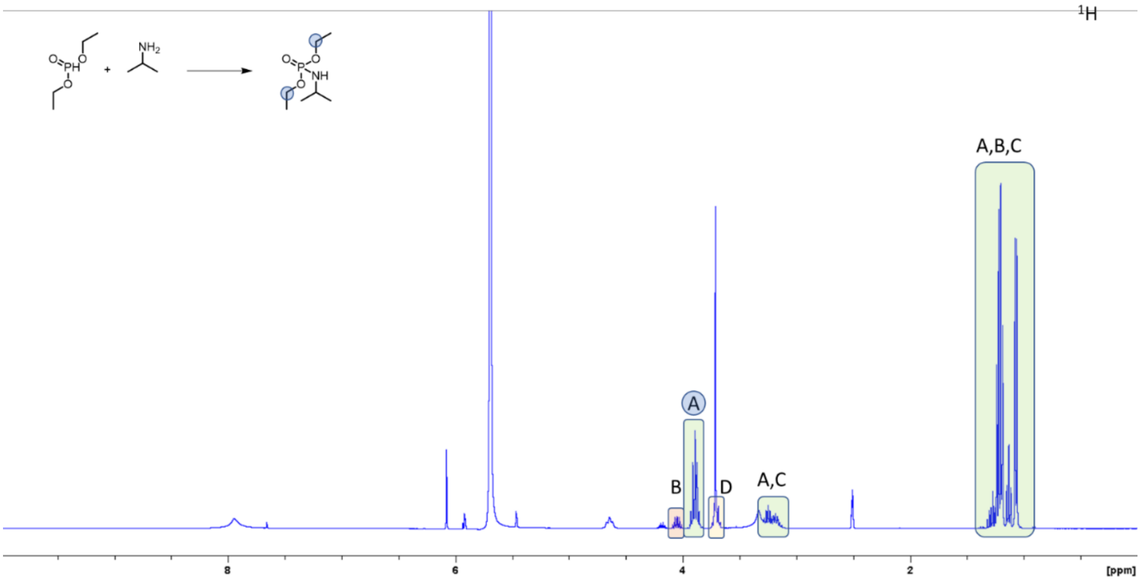

A: Diethyl isopropylphosphoramidate, B: Diethyl phosphite, C: Isopropylamine, D: Diethyl phosphate

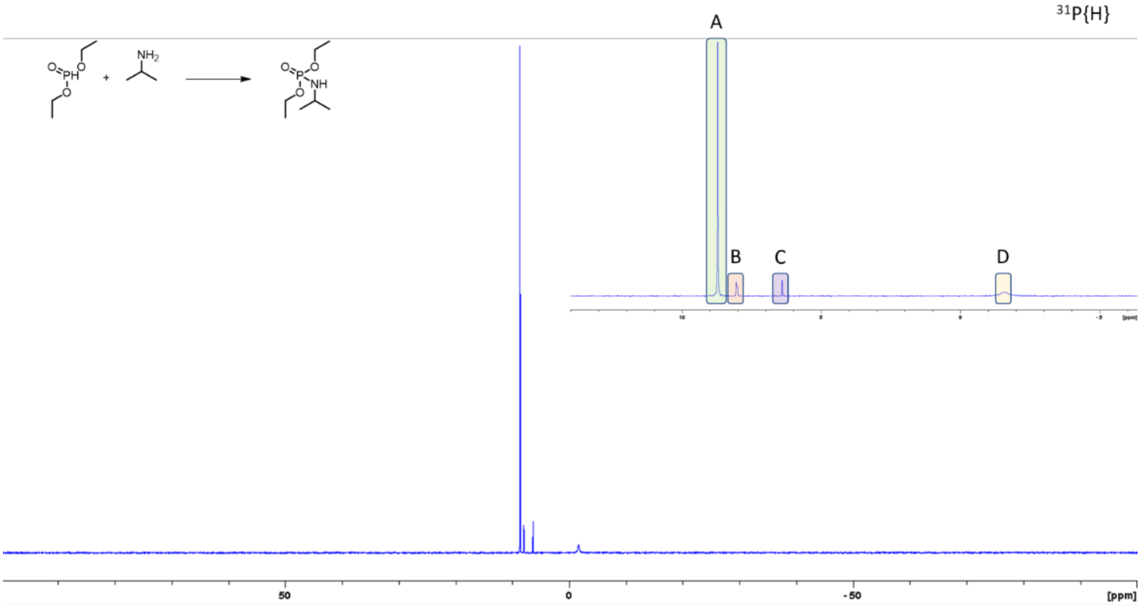

A: Diethyl isopropylphosphoramidate, B: Diethyl phosphite, C: Unknown, D: Diethyl phosphate

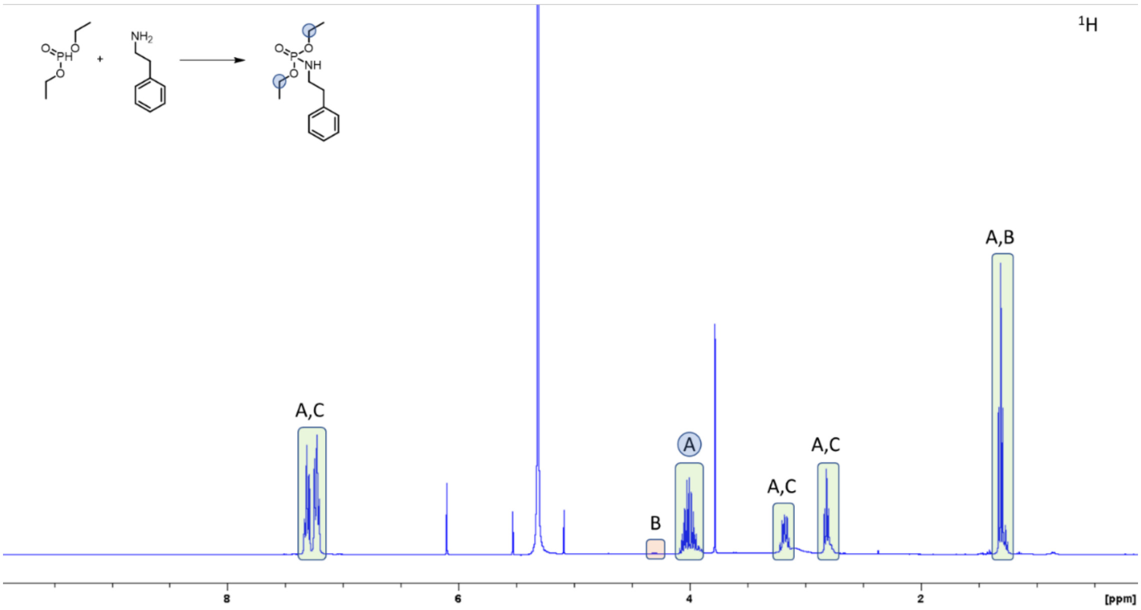

A: Diethyl phenylethylphosphoramidate, B: Diethyl phosphite, C: Phenylethylamine

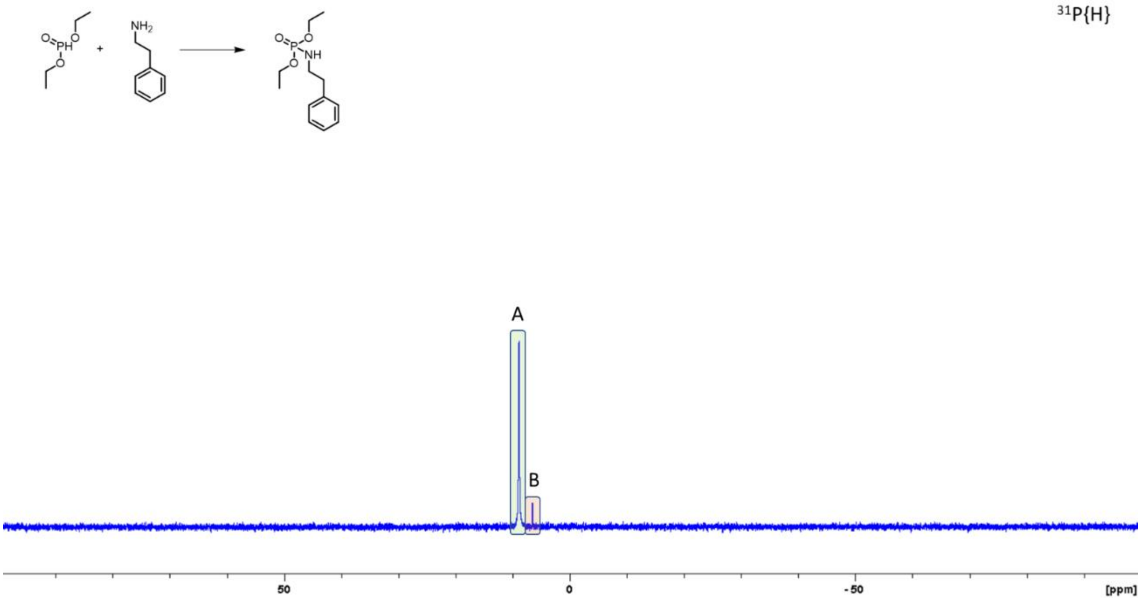

A: Diethyl phenylethylphosphoramidate, B: Diethyl phosphite

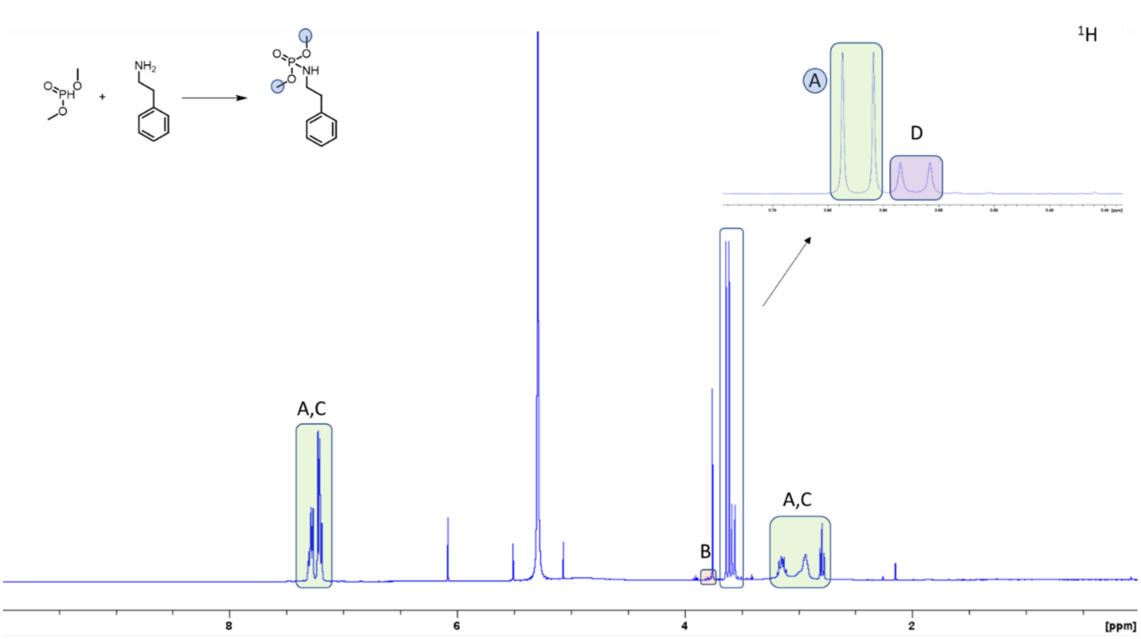

A: Dimethyl phenylethylphosphoramidate, B: Dimethyl phosphite, C: Phenylethylamine, D: Dimethyl phosphate

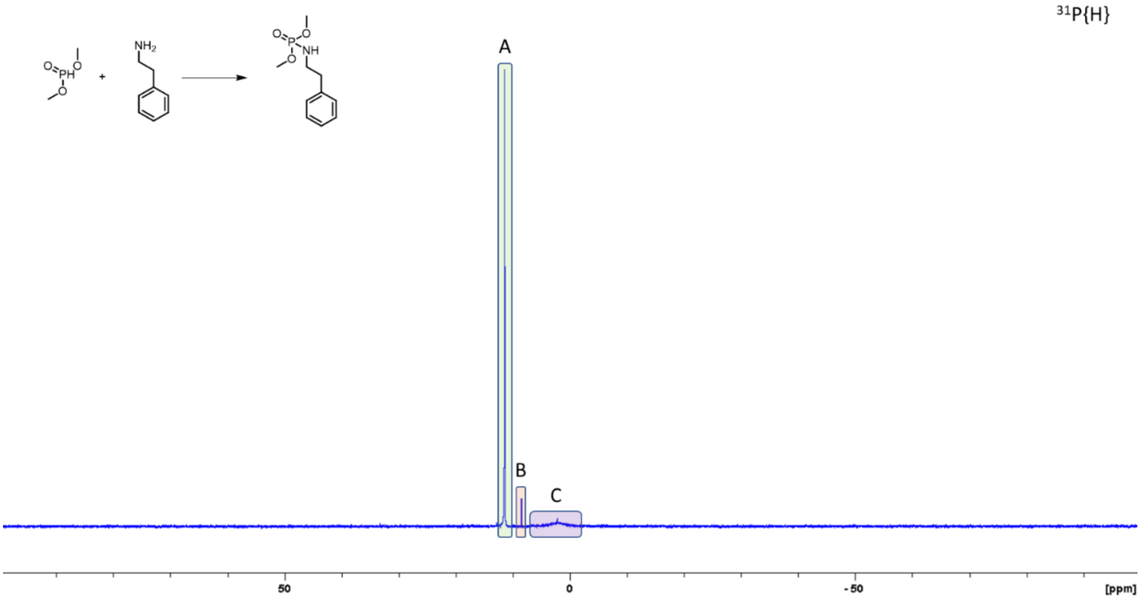

A: Dimethyl phenylethylphosphoramidate, B: Dimethyl phosphite, C: Dimethyl phosphate

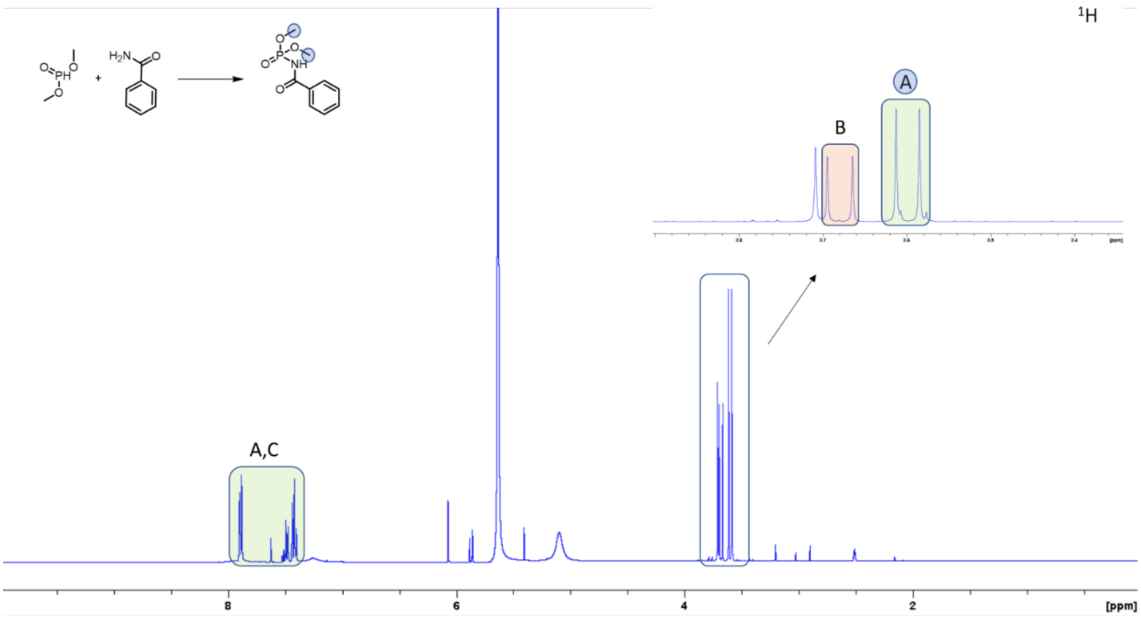

A: Dimethyl benzoylphosphoramidate, B: Dimethyl phosphite, C: Benzamide

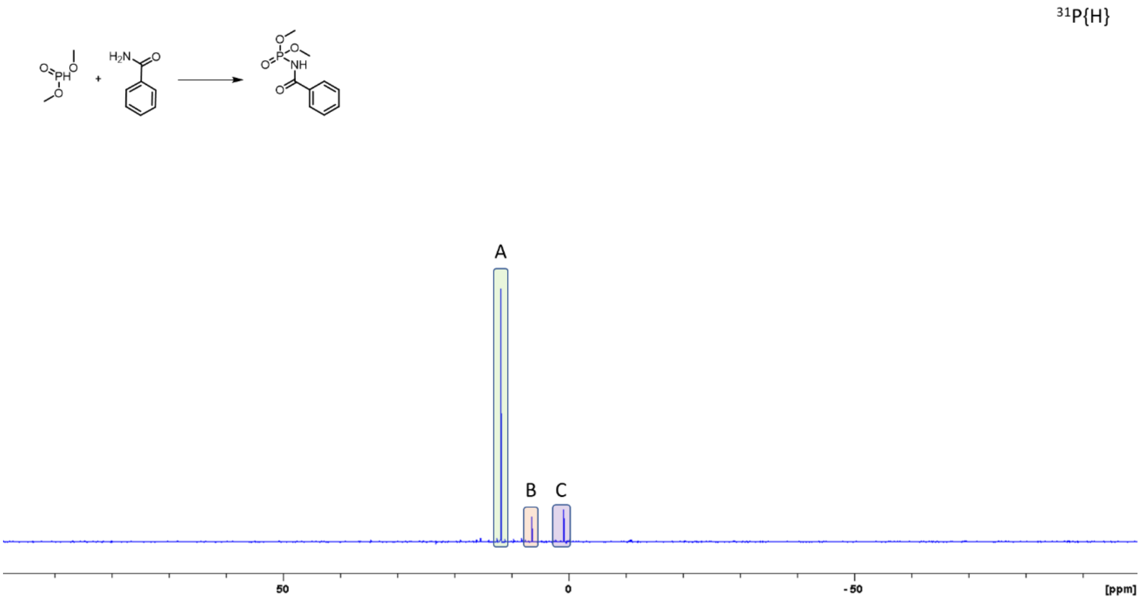

A: Dimethyl benzoylphosphoramidate, B: Dimethyl phosphite, C: Unknown

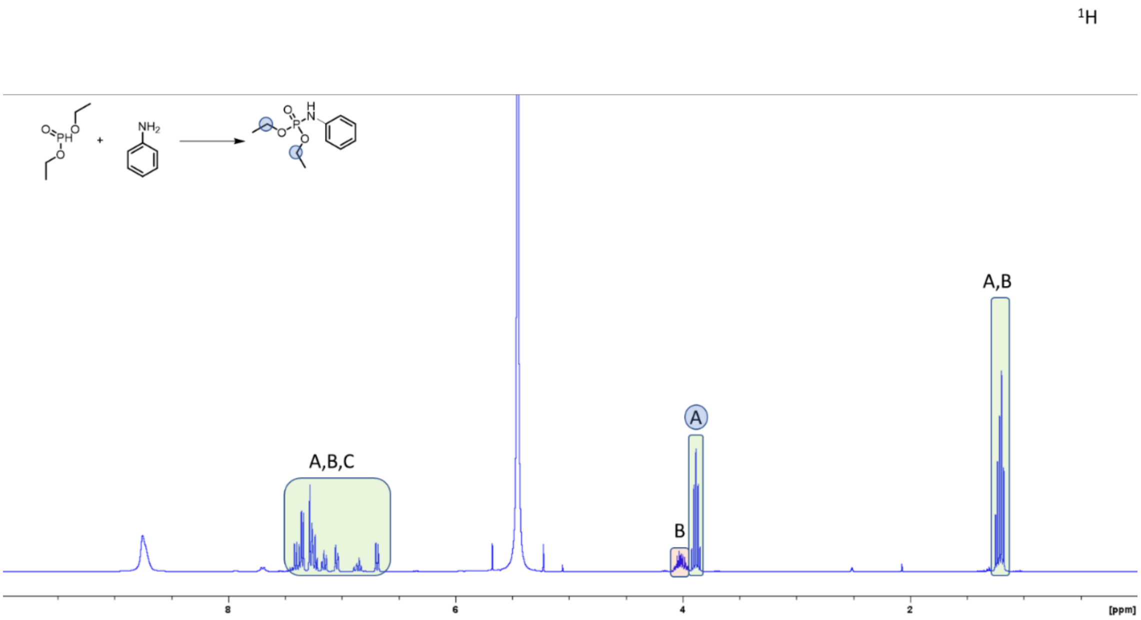

A: Diethyl phenylphosphoramidate , B: Diethyl phosphite, C: Aniline

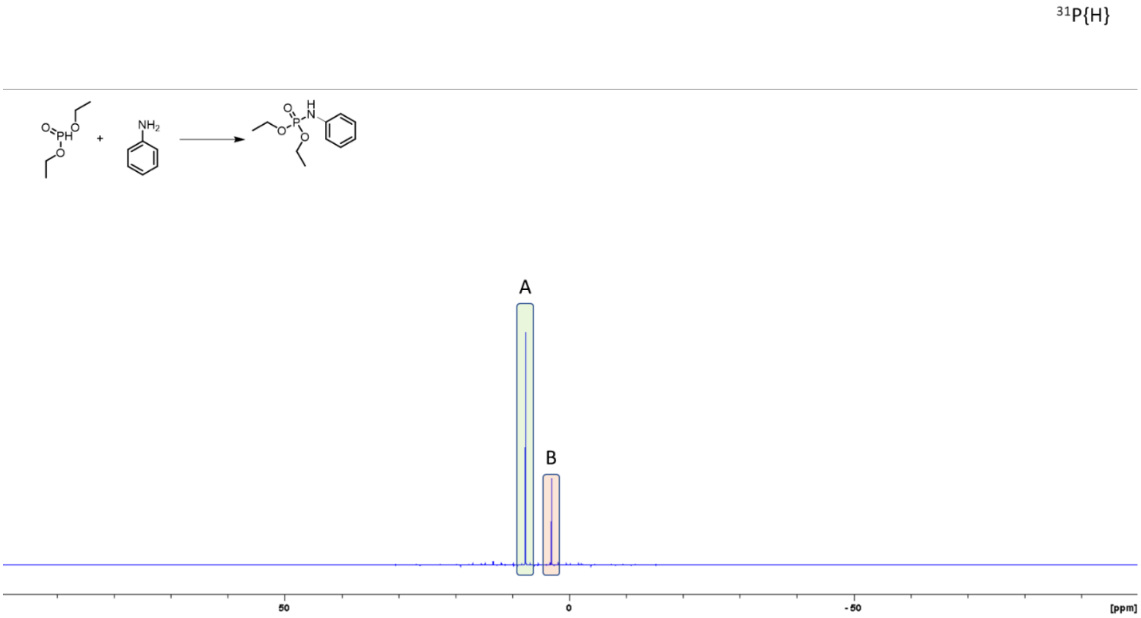

A: Diethyl phenylphosphoramidate , B: Diethyl phosphite

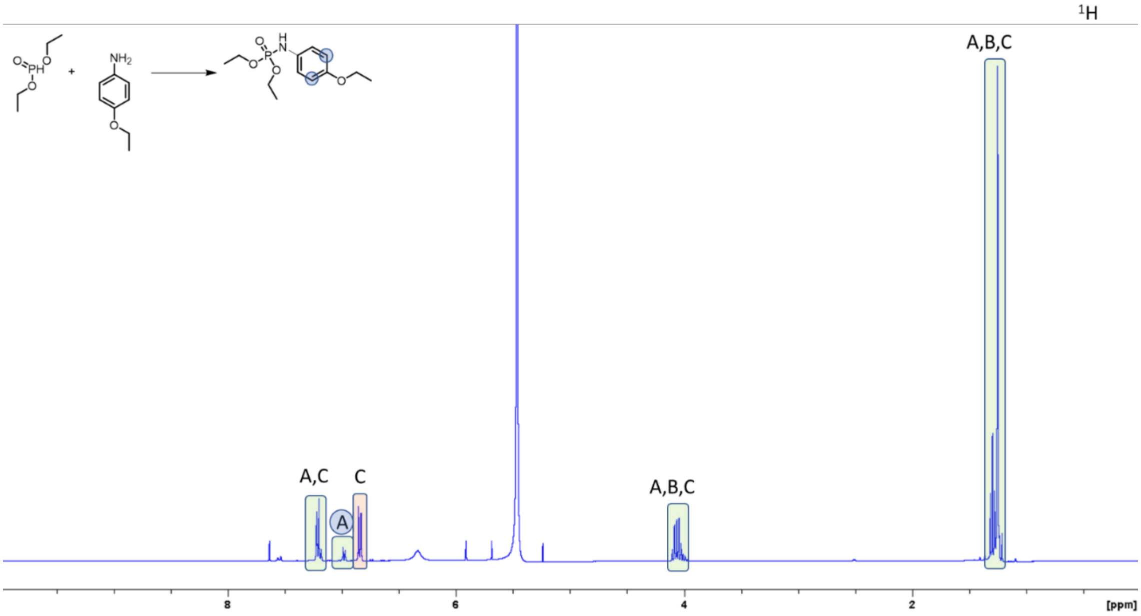

A: Diethyl (4-ethoxyphenyl)phosphoramidate , B: Diethyl phosphite, C: 4-Ethoxyaniline

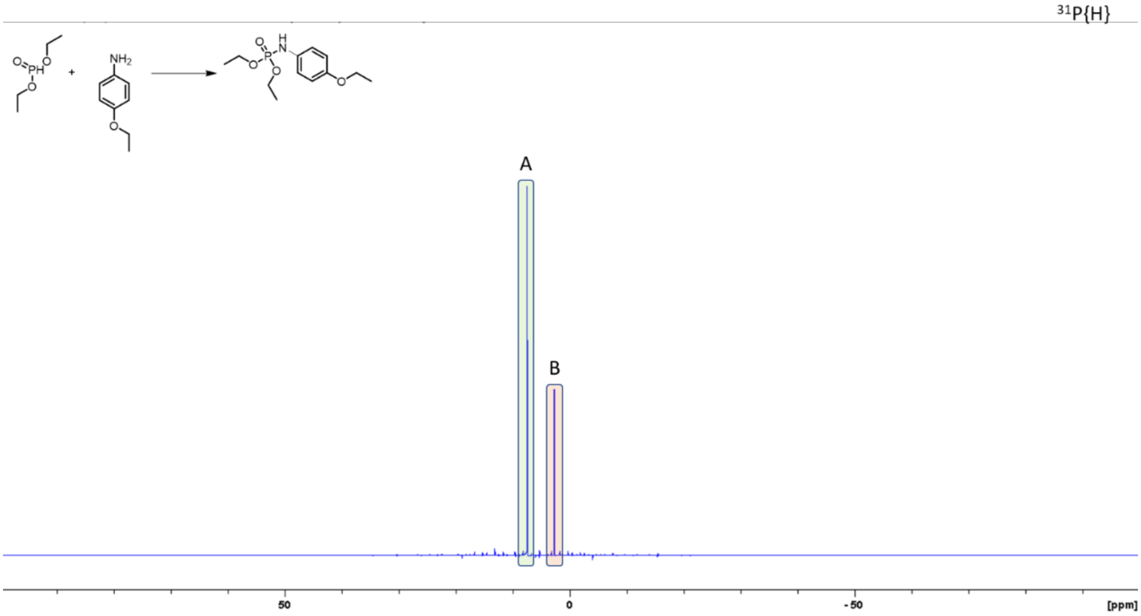

A: Diethyl (4-ethoxyphenyl)phosphoramidate , B: Diethyl phosphite

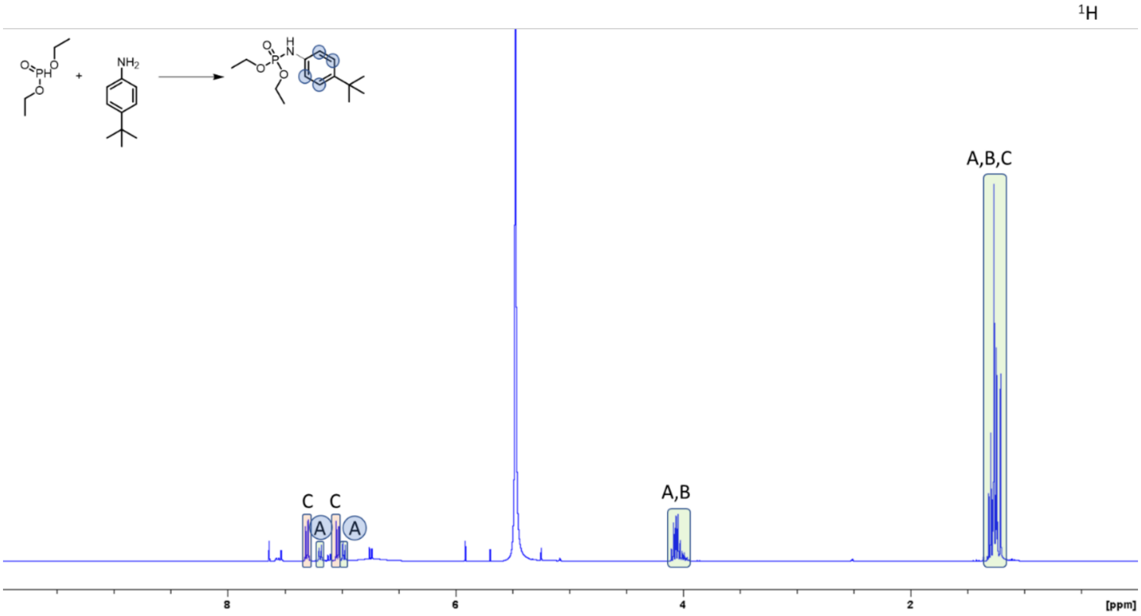

A: Diethyl 4(*tert*-butyl)phosphoramidate , B: Diethyl phosphite, C: 4-*tert*-Butylaniline

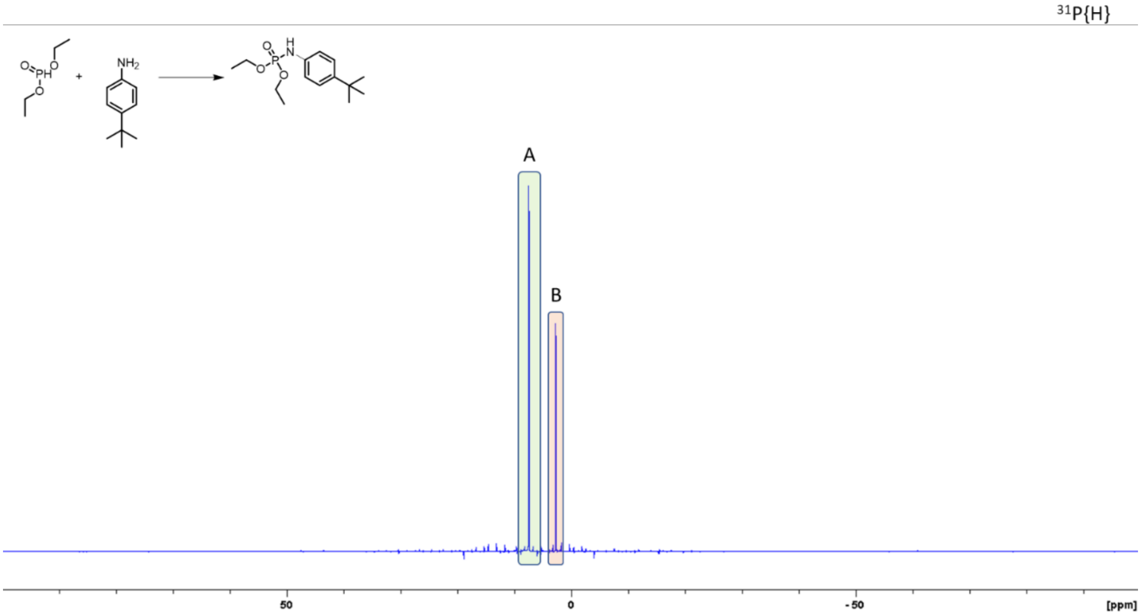

A: Diethyl 4(*tert*-butyl)phosphoramidate , B: Diethyl phosphite,

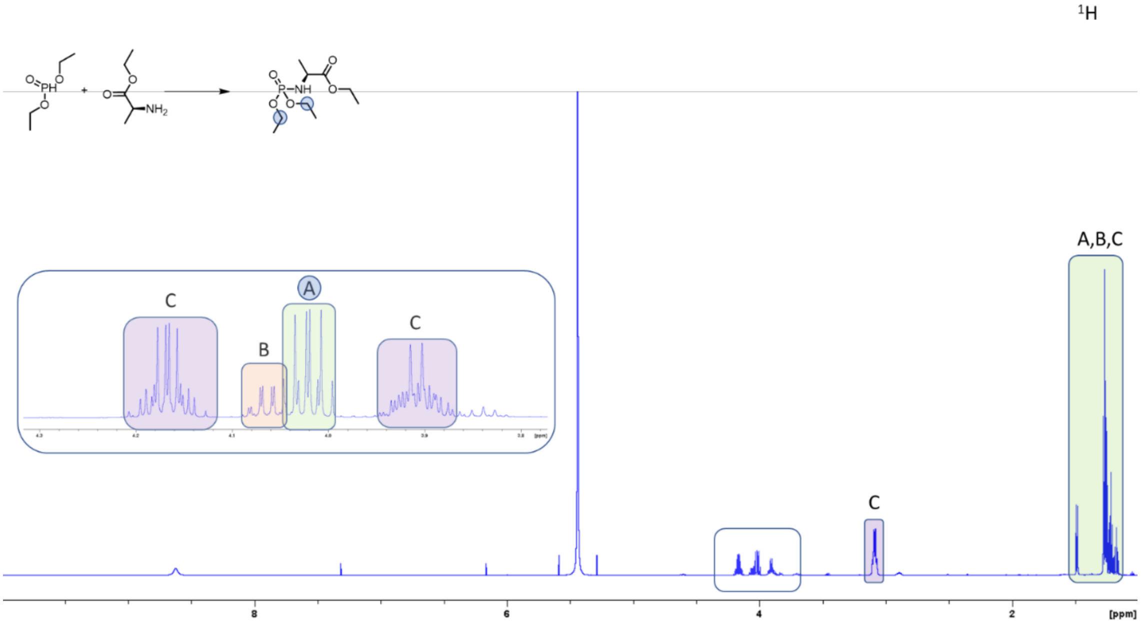

A: Ethyl (diethoxyphosphoryl)-L-alaninate, B: Diethyl phosphite, C: L-Alanine ethyl ester

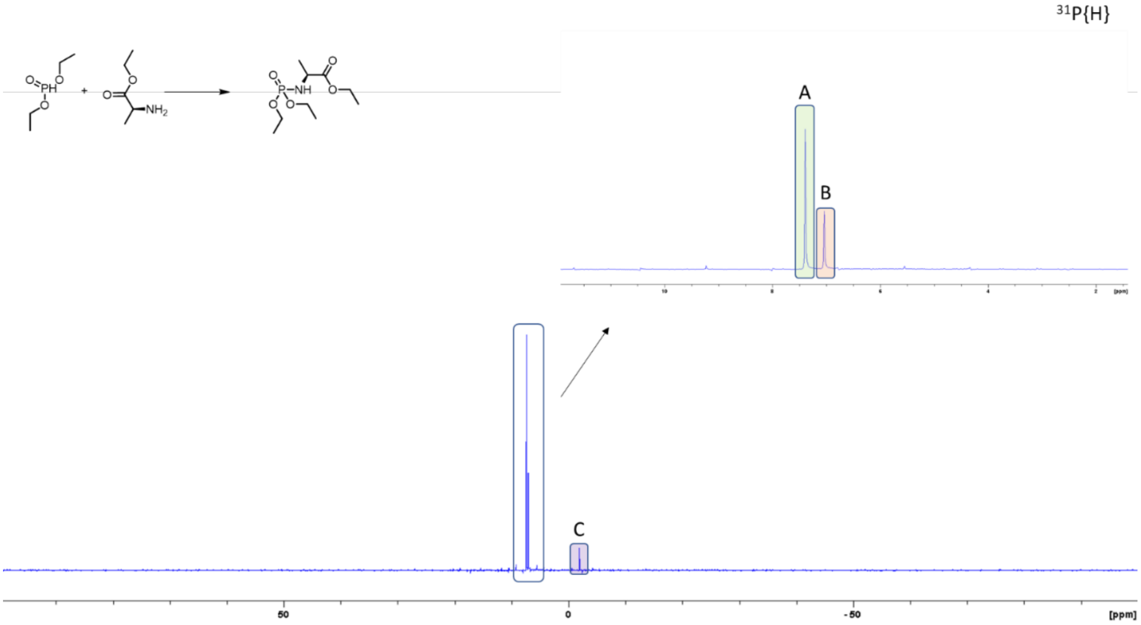

A: Ethyl (diethoxyphosphoryl)-L-alaninate, B: Diethyl phosphite, C: Diethyl phosphate

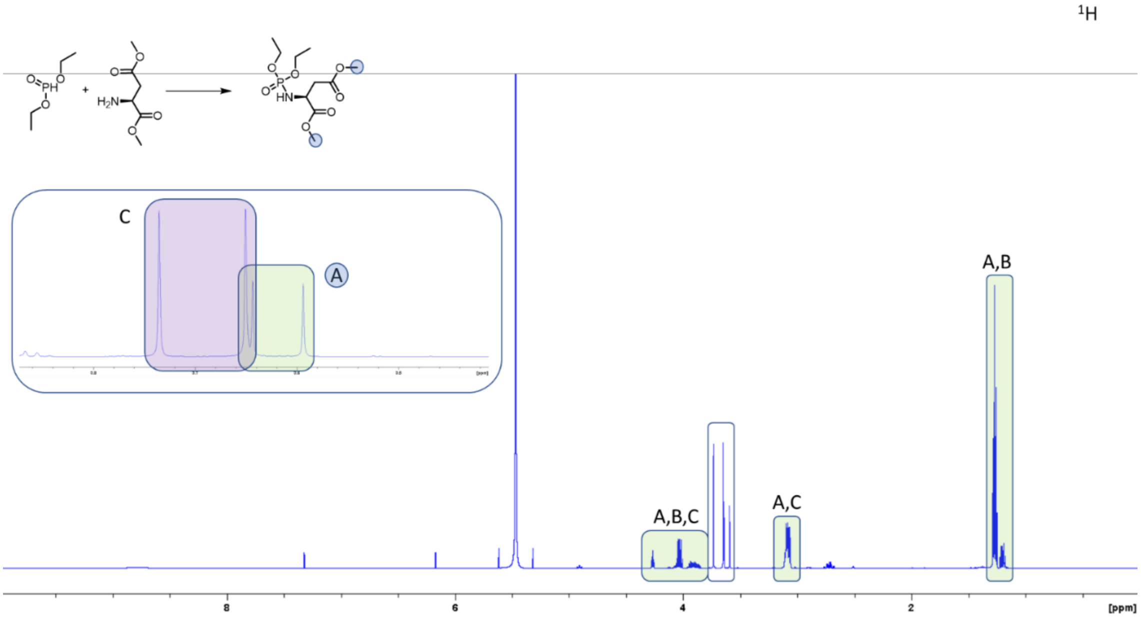

A: Dimethyl (diethoxyphosphoryl)-L-aspartate, B: Diethyl phosphite, C: Dimethyl L-Aspartate

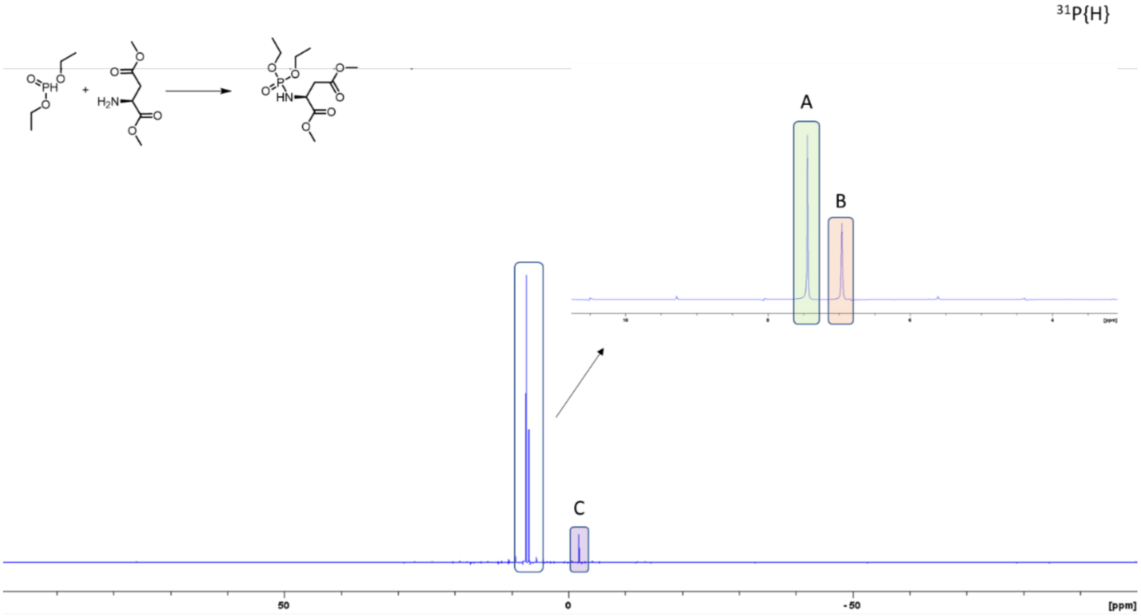

A: Dimethyl (diethoxyphosphoryl)-L-aspartate, B: Diethyl phosphite, C: Diethyl phosphate

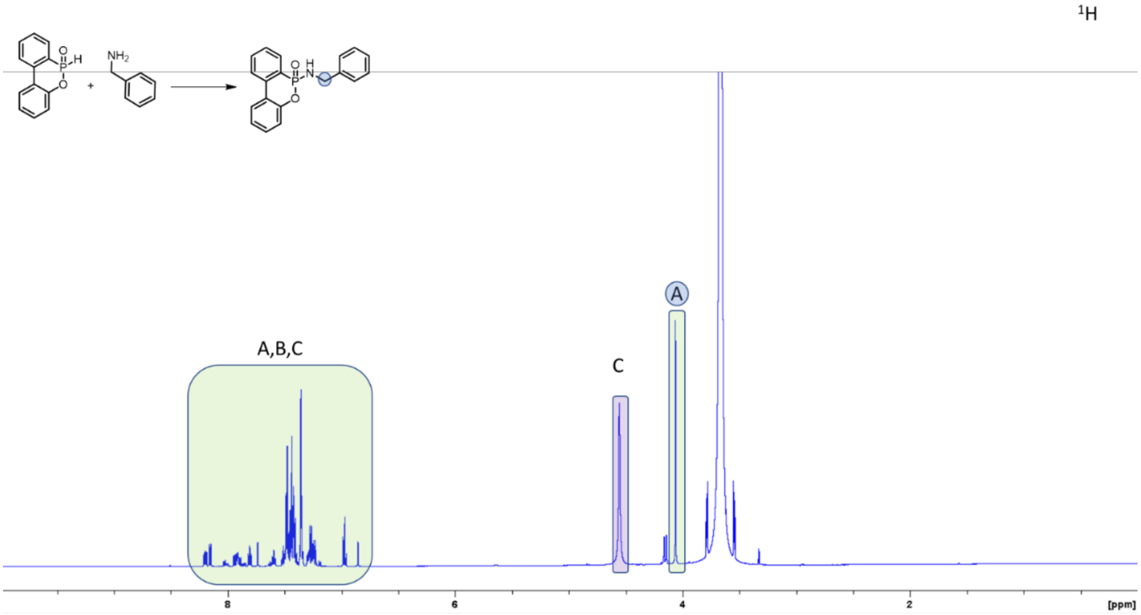

A: 6-(benzylamino)dibenzo[c,e][1,2]oxaphosphinine 6-oxide, B: dibenzo[c,e][1,2]oxaphosphinine 6-oxide, C: benzylamine

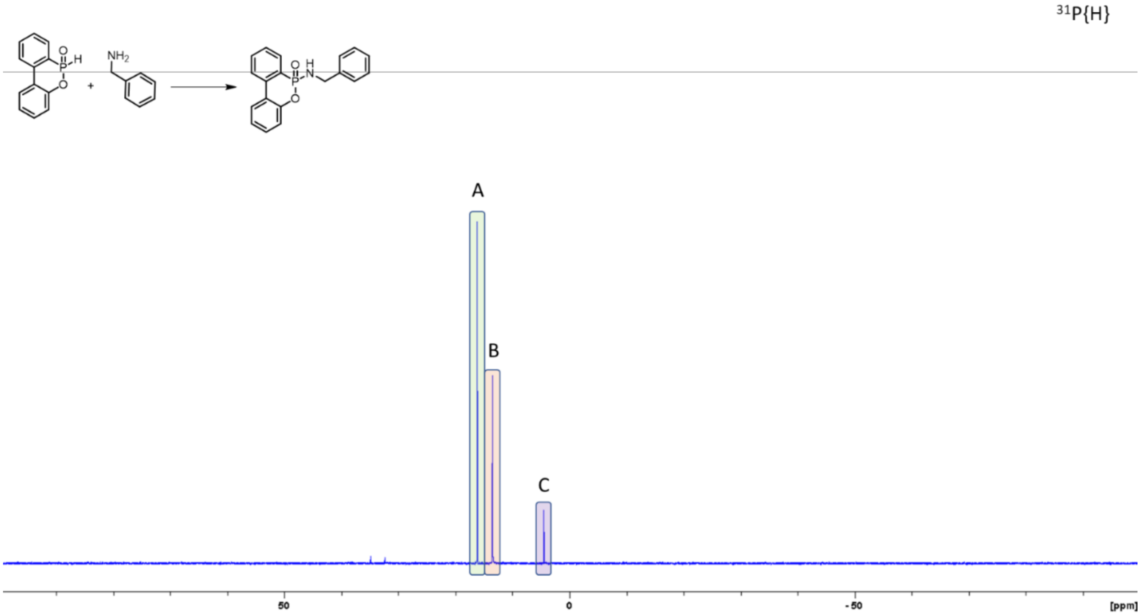

A: 6-(benzylamino)dibenzo[c,e][1,2]oxaphosphinine 6-oxide, B: dibenzo[c,e][1,2]oxaphosphinine 6-oxide, C: 6-hydroxydibenzo[c,e][1,2]oxaphosphinine 6-oxide
